# Shifts in biodiversity and physical structure of seagrass beds across 5 decades at Carriacou, Grenadines

**DOI:** 10.1371/journal.pone.0306897

**Published:** 2024-08-01

**Authors:** David Patriquin, Robert E. Scheibling, Karen Filbee-Dexter

**Affiliations:** 1 Department of Biology, Dalhousie University, Halifax, Nova Scotia, Canada; 2 University of Western Australia, Perth, Australia; 3 Institute of Marine Research, His, Norway; The University of Auckland - City Campus: University of Auckland, NEW ZEALAND

## Abstract

Caribbean seagrass beds are facing increasing anthropogenic stress, yet comprehensive ground-level monitoring programs that capture the structure of seagrass communities before the 1980s are rare. We measured the distribution of seagrass beds and species composition and abundance of seagrass and associated macroalgae and macroinvertebrates in 3 years over a 47-year period (1969, 1994, 2016) at Carriacou, Granada, an area not heavily impacted by local human activity. Seagrass cover and physical parameters of fringing beds were measured in transects at high (HWE) and low wave energy (LWE) sites; frequency of occurrence of all species, and biomass and morphology of seagrasses, were measured at 100 m^2^ stations around the island. Losses in nearshore seagrass cover occurred at HWE but not LWE sites between 1969 and 2016 and were associated with increases in the seagrass-free inshore zone (SFI) and erosional scarps within beds. Total biomass did not vary across years although there were progressive changes in seagrass composition: a decline in the dominant *Thalassia testudinum* and concomitant increase in *Syringodium filiforme*, and establishment of invasive *Halophila stipulacea* in 2016 at LWE sites. Species richness and diversity of the seagrass community were highest in 1994, when 94% of macroalgae (excluding *Caulerpa*) were most abundant, and sea urchins were least abundant, compared to 1969 and 2016. Multivariate statistical analyses showed differences in community composition across the 3 years that were consistent with trends in urchin abundance. Increases in SFI and scarp number in seagrass beds at HWE sites occurred mainly after 1994 and likely were related to increased wave forcing following degradation of offshore coral reefs between 1994 and 2016. Our observations suggest that landward migration of seagrass beds with rapidly rising sea level in future will not be realized in reef-protected seagrass beds at Carriacou barring reversal in the processes that have caused reef flattening.

## Introduction

Seagrasses are major components of coastal ecosystems in the Tropical Atlantic Bioregion [1]. They colonize rubble, sand or muddy substrates on reef flats, back-reef lagoons, and in sheltered bays and estuaries. Seagrass beds provide significant ecosystem services locally as nurseries and feeding areas for fish on coral reefs and mangroves [2–4], and as habitat for traditionally fished sea urchins [5], green turtle (now protected) [6], conch [7] and lobster [8]. By dampening wave action and stabilizing sediments, seagrasses enhance coastal protection and water quality [9–11] and calcareous epiphytes and seaweeds in seagrass beds are significant contributors to sediments [12]. Interactions between seagrass habitats and nearby coral reefs and mangroves increase overall health of various habitat types [10, 13]. Seagrasses also counteract ocean acidification locally by raising pH leading to significantly increased calcification by corals downstream of seagrass beds [14] and by calcareous algae within beds [15]. Seagrasses, like mangroves and saltmarshes, are significant contributors to blue carbon [16] due to their high productivity and belowground carbon storage [17]. However, there remains considerable uncertainty about the total area of seagrass beds globally [18], and carbon storage varies widely according to site conditions and seagrass species [19].

Recent estimates [20] indicate that seagrass cover globally has declined by 19% since the 1880s, with declines occurring in all seven of seagrass bioregions defined by [1]. Absolute losses were highest in the Tropical Atlantic Bioregion, although there has been a trend of increase in seagrass cover in this region since the late 1990s. Principal drivers of sustained losses globally, and in the Tropical Atlantic Bioregion specifically, are coastal development and poor water quality [20–22]. Increases in seagrass cover have been observed in some areas without any management [23] and others after deliberate actions such as reduction of nutrient input [24]. In general, seagrass beds in the Caribbean have proved remarkably resilient to extreme storms, either being minimally impacted (especially those dominated by the climax species *Thalassia testudinum*) or recovering over time [11, 21]. However there have been shifts towards earlier successional seagrass species, and disruptions by intense storm activity appear to facilitate expansion of *Halophila stipulacea* [25], an invasive seagrass first noted in the Tropical Atlantic at Grenada in 2002 [26]. Such changes can affect ecosystem services such as coastal protection [27].

Sea level rise can have negative or positive effects on the area of seagrass and other coastal wetlands depending on their capacity to build vertically, sediment supply, and the lateral and vertical “accommodation space” for landward migration of wetlands, and extent to which this is realized through local coastal management [19, 28, 29]. Global mean sea level rise for the Caribbean, estimated as 2.8 mm yr^−1^ for 1993–2018, is predicted to rise dramatically after 2050, increasing to as much as 30 mm yr^−1^ by 2100 [30]. Such increases could lead to disproportionally large increases in wave energy and negative impacts on seagrass beds in back-reef environments if vertical reef accretion does not keep pace [31, 32]. Reef “flattening”, the loss of hard coral and reef structural complexity, has been widespread in the Caribbean since the early 1980s [33]. Based on modelling studies [34], showed that a significant increase in mean wave height could occur at present sea level given sustained degradation of reef structural complexity.

Observations conducted on seagrass beds at Carriacou, Grenada in the eastern Caribbean in 1969, 1994 and 2016 provide an opportunity to explore changes in seagrass distribution and community composition on a multidecadal scale in an area not heavily impacted by local human activity aside from subsistence fishing. These seagrass beds occur in windward lagoons behind barrier reefs as well as in leeward areas of the island [35, 36]. The barrier reefs have undergone the types of degradation [37] observed more generally for the Caribbean [38, 39]. The initial set of observations in 1969 was conducted as part of a broad characterization of *T*. *testudinum* and “turtle grass beds” at Barbados and Carriacou in the eastern Caribbean [35, 40]. Subsets of those observations were repeated at Carriacou, the less developed island, in 1994 and 2016. Scheibling et al. [36] reported on the distribution and abundance of the invasive seagrass *H*. *stipulacea* at these and other sites in Carriacou in 2016.

The primary objectives of our study are:

1. To compare changes in abundance (cover, frequency of occurrence, biomass, shoot density) and morphology of *T*. *testudinum*, considered the dominant or climax species at the onset of our study, along with changes in abundance of other native or invasive seagrass species and of associated macroalgae and sessile invertebrates (corals, sponges) across this 47-year period.
2. To use a species-level measure of abundance (frequency of occurrence) to examine temporal changes in biodiversity and community composition of seagrass beds.
3. To provide concurrent measures of physical parameters of seagrass beds (extent of the seagrass-free inshore zone, depth profiles and frequency of erosional scarps) that can be affected by changing hydrodynamic forces, and to relate these to population and community level changes documented in objectives 1 and 2.

At this time of accelerating climatic change and increasing levels of global and local human impact on coastal ecosystems, the importance of establishing baselines against which ecological change can be measured becomes increasingly apparent. Longitudinal studies are particularly valuable in establishing relevant baselines or in providing some semblance of a pristine state, that enable comparisons across geographic regions and inform conservation and management initiatives [41]. Most comparisons of seagrass systems over periods of 40 years or more are based on aerial imagery [20, 23]. Comprehensive monitoring programs that capture ground-level observations on the structure of tropical seagrass communities before the 1980s are rare [22]. Our study provides this insight for a relatively undeveloped location in the Caribbean that has nonetheless been impacted by broad-scale changes in biotic and abiotic factors that have shaped the local seagrass ecosystem.

## Materials and methods

### Study sites and sampling methods

To provide a basic description of “turtle grass beds” (seagrass beds dominated by *Thalassia testudinum*) around Carriacou in 1969, areas putatively dominated by *T*. *testudinum* were identified as dark patches in aerial photographs of the island taken in 1966 by the Directorate of Overseas Surveys, United Kingdom (Contract 85, print scale 1:12,900). Detailed tracings of these patches provided cursory maps of seagrass distribution that were used to select study sites representative of seagrass beds around the island. For logistical reasons, most sites were on the east, windward coast. Sites were located by reference to prominent geographic features (e.g., promontories, beaches, cays, reefs) and accessed directly from shore or with a small motorboat. As a measure of ground-truthing, *T*. *testudinum* was present at all putative turtle grass sites during the initial sampling in 1969. Sampling took place in March 1969, late January to early March 1994, and January to February 2016 (except 1 transect in February 2019). Sampling was mainly done by snorkeling; SCUBA was used to access deeper beds in some offshore regions in 1994.

### Transects

**Fig 1:**
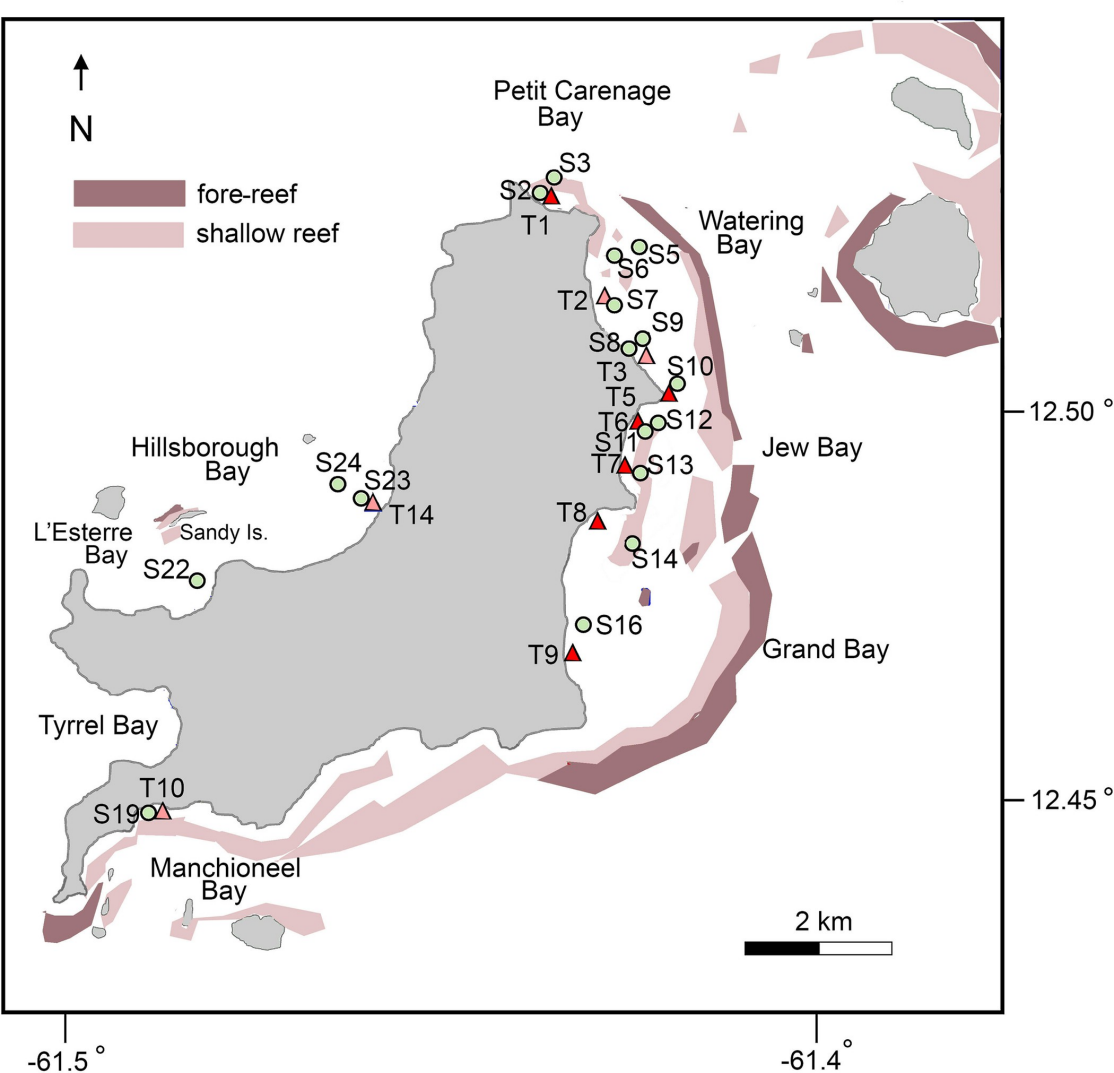
Sites where seagrass beds were sampled at Carriacou. Sampling stations (S) were distributed across representative seagrass beds around the island (green circles); transects (T) were sampled at low wave energy (orange triangles) and high wave energy (red triangles) sites. See S1 Table for GPS coordinates and additional site description. Also shown is a diagrammatic representation of coral reefs around Carriacou and surrounding islands, after [37].

Eleven sites were selected in 1969 to sample line transects that spanned fringing seagrass beds around Carriacou (i.e., located along the shoreline) [35]. Ten sites were resampled in 1994 and 2016 (Fig 1, S1 Table); one of the 1969 line transects could not be relocated. Transects were run from shore (sea level), and perpendicular to it, across all or part of a fringing bed. Transect length in 1969 was variable [35]; transects extended to at least the point where no further erosional scarps occurred, confirmed by observation well beyond the last scarp. Direction was maintained using a compass or by reference to two aligned markers on the shore. Transects were conducted within 1–2 h of low tide when low current and high visibility optimized sampling. Distances and depths along transects were measured with a calibrated 2-m pole sequentially flipped in the horizontal direction and vertically positioned at 10-m intervals, or less where there were abrupt changes in depth; a depth gauge was used for depths > 2 m. Depth was adjusted to mean low water (MLW). The presence of different seagrass species, and of seagrass-free bottom, were recorded at points (distances) along transects where changes in seagrass composition occurred. The recorded substratum cover type was deemed constant from each observation point until the point where there was a change in type. Total cover per substratum type was calculated as the sum of the distances (in m) represented by that type along a transect to 50 m offshore, common to all transects (and thus restricted to nearshore areas only). Cover values were converted to percent of the total distance (50 m) for analysis.

We counted erosional scarps occurring on the inshore margin of seagrass beds and associated with “blowouts” within the seagrass beds [35] along transects, including those beyond 50 m. Only actively eroding scarps (i.e. those with underlying seagrass bed sediments exposed) were counted; older, overgrown scarps were not included in these counts. We also measured the extent of the seagrass-free inshore zone (SFI) as the distance along a transect (in m) from the shore at MLW to the first occurrence of seagrass. This zone often was the subtidal extension of a sandy beach; in some areas it contained scattered rubble, boulders or sparse macroalgae but no coherent seagrass patch or bed. We limited our analysis of these erosional features to 80 m offshore, common to all transects.

In 1994, due to logistical constraints, the full set of transect measurements was limited to 4 of the 10 transects (T6, T7, T9, T14); scarps however were counted on all 10 transects. In 2016, the full set of transect measurements was repeated on 9 the 10 transects. The remaining transect (T9) was incorrectly positioned in 2016; the correct site was sampled in February 2019 and the observations included with the 2016 data.

Based on observed wave conditions in the field, we classified 6 transects (T1, T5 –T9) as HWE (High Wave Energy) sites, and 4 transects T2, T3, T10 and T14 as LWE (Low Wave Energy) sites in 1969. Relative Exposure Index was calculated after [42, 43] for the shore position on each of the 10 transects in 2016 (2019 for T9) based on local wind data [44] and limiting ocean fetch to 200 km [45]. There was an order of magnitude difference in mean REI between HWE sites (T1, T5 –T9: 5327) and LWE sites (T2, T3, T10, T14: 421) in 2016 (S1 Fig), consistent with our qualitative classification. The minimum REI recorded at 1 HWE site (T1) was equivalent to the maximum recorded at 1 LWE Site (T2). However, our observations of wave conditions at T1 indicated it was a high wave energy site, which was not captured in the REI due to the reduction in fetch from surrounding islands. This HWE designation was supported by the occurrence of coarser, better sorted sediments at T1 than T2 based on samples in 1969 [35]. Based on studies of reef sediment transport and distribution off the east coast of Carriacou, Watering Bay (where T2 is located) has been characterized as a low wave energy environment [46, 47].

### Stations

In addition to line transect sites, in 1969, 24 sampling stations (10 x 10 m grids) were located at sites chosen to be representative of seagrass beds around Carriacou, as viewed in the aerial photographs (e.g., fringing bed, offshore patch, lagoonal patch, cobble bank). Of these 24 stations, 17 were resampled in both 1994 and 2016 and form the basis of our spatial-temporal comparisons (Fig 1, S1 Table). At each station, 12 quadrats (0.25 m^2^) were randomly positioned within the sampling grid. Presence of all readily visible macroflora (seagrasses, seaweeds) and macrofauna (sponges, corals, echinoderms, conch) was recorded in each quadrat. In 1969 and 1994, macroalgal specimens that were not clearly identifiable in the field were collected for subsequent description and identification. Macroalgae were identified by reference to Taylor [48]. A collection of 14 sponges in 1969 was identified by Prof. George Hechtel (State University of New York) in 1971. In 2016, photographs of all species were taken with an Olympus TG4 camera and placed on iNaturalist (www.inaturalist.org/projects/seagrass-bed-flora-fauna-of-carriacou-grenada-2016). In the final compilation of species occurrence for comparison between the 3 sampling years, 69 species or “species groups” could be reliably compared between the 3 sampling years. These include 3 seagrasses, 46 macroalgae, 2 echinoderms, 1 gastropod, 6 corals, 7 sponges and 4 gorgonians. Of these, 56 were identified to the species level. The remaining 13 were classified into “species groups”, which included closely related species that were not reliably or consistently distinguished in the field or for which the taxonomic distinctions are not well defined (e.g., the *Porites furcata* group refers to *P*. *furcata* and/or *P*. *divaricata*; *Tripneustes ventricosus* group refers to *T*. *ventricosus* and *Lytechinus variegatus*).

In 1969, 1994 and 2016, samples of seagrass (cut at substrate level) were collected from 3 haphazardly placed 0.0625 m^2^ quadrats within each 10 x 10 m sampling station. Seagrasses were sorted by species, shaken to remove excess water, and weighed fresh as a measure of above ground biomass. For *T*. *testudinum*, leaf width was measured (0.5 mm precision) for 35 haphazardly sampled leaves. Maximum leaf length was calculated as L_10_, the average length (0.5 cm precision) of the longest 10 leaves for samples of 200 leaves and greater, or as L_5%_, the average length of the 5% of leaves for samples with less than 200 leaves [49]. In 1994 and 2016, we also counted the number of leaves of *T*. *testudinum* in our biomass samples, and the number of leaves per shoot for 25 shoots sampled *in situ* at each station. Shoot density was estimated by dividing the total number of leaves per unit area by the average number of leaves per shoot. We used a significant linear relationship (F_1,30_ = 179, P < 0.001) between mass per leaf and the product of maximum length and average width, based on our 1994 and 2016 samples (when all leaves were measured and counted), to estimate leaf number for the 1969 samples from the 1969 measures of maximum leaf length, average leaf width and total fresh weight. We used the average number of leaves per shoot for sites sampled in 1994 to estimate shoot densities in 1969. This assumes that the number of leaves per shoot is similar between years, consistent with our observations in 1994 and 2016 (see Results).

### Ethics statement

Aside from sponge samples collected for species identification in 1969, no other physical samples of macrofauna were taken and no permits were required. Throughout the study, sites were accessed mainly by sea and not on privately owned or protected land. No protected species were sampled, and there was no field or laboratory experimentation.

### Statistical analyses

Differences in frequency of occurrence and biomass of seagrass species across years were compared using 1-way ANOVA for each species and response variable. Shoot density and leaf length and width for *T*. *testudinum* were compared using 1-way ANOVA for each response variable. Differences in scarp number across wave exposure and year was compared using a 2-way fixed effects ANOVA (car package) with type III sums of squares for unbalanced design.

We examined patterns of community diversity using community characteristics (species richness, Shannon diversity) calculated for each station from frequency of occurrence data (proportional frequency over the 12 quadrats) restricted to the 66 species that formed the sessile benthic assemblage; mobile species (*Tripneustes ventricosus* group, *Oreaster reticulatus*, and *Aliger gigas*) were excluded. Differences in these response variables across years were tested using 1-way ANOVA. Assumptions of homogeneity of residual variance were evaluated using Levene’s test and inspection of plotted residuals. Where we detected significant main effects, posthoc tests were performed using Tukey’s HSD.

We examined the assemblage structure for all three years and all stations using multivariate analysis. Difference between years were analyzed using a one factor permutational multivariate analysis of variance (PERMANOVA) based on a Bray–Curtis similarity matrix generated from arcsine sqrt transformed data [50]. Year was a fixed factor and analysis used type III sums of squares. Non-metric multidimensional scaling (nMDS) ordination, based on the same Bray–Curtis similarity matrix, was used to examine multivariate patterns in the composition of the benthic assemblage among years and stations. Spearman’s rank correlation was used to examine relationships between ordination scores and the frequency of occurrence of main functional groups (green algae, red algae, brown algae, corals, and seagrass). To test the hypothesis that variation in the sessile benthic community was related to variation in sea urchin abundance, we also used Spearman’s rank correlation for the ordination scores and examined the frequency of occurrence of *Tripneustes ventricosus* at each station. All multivariate procedures reported here were performed using Primer 7 with the PERMANOVA add-on [51].

## Results

### Seagrass cover on transects

**Fig 2:**
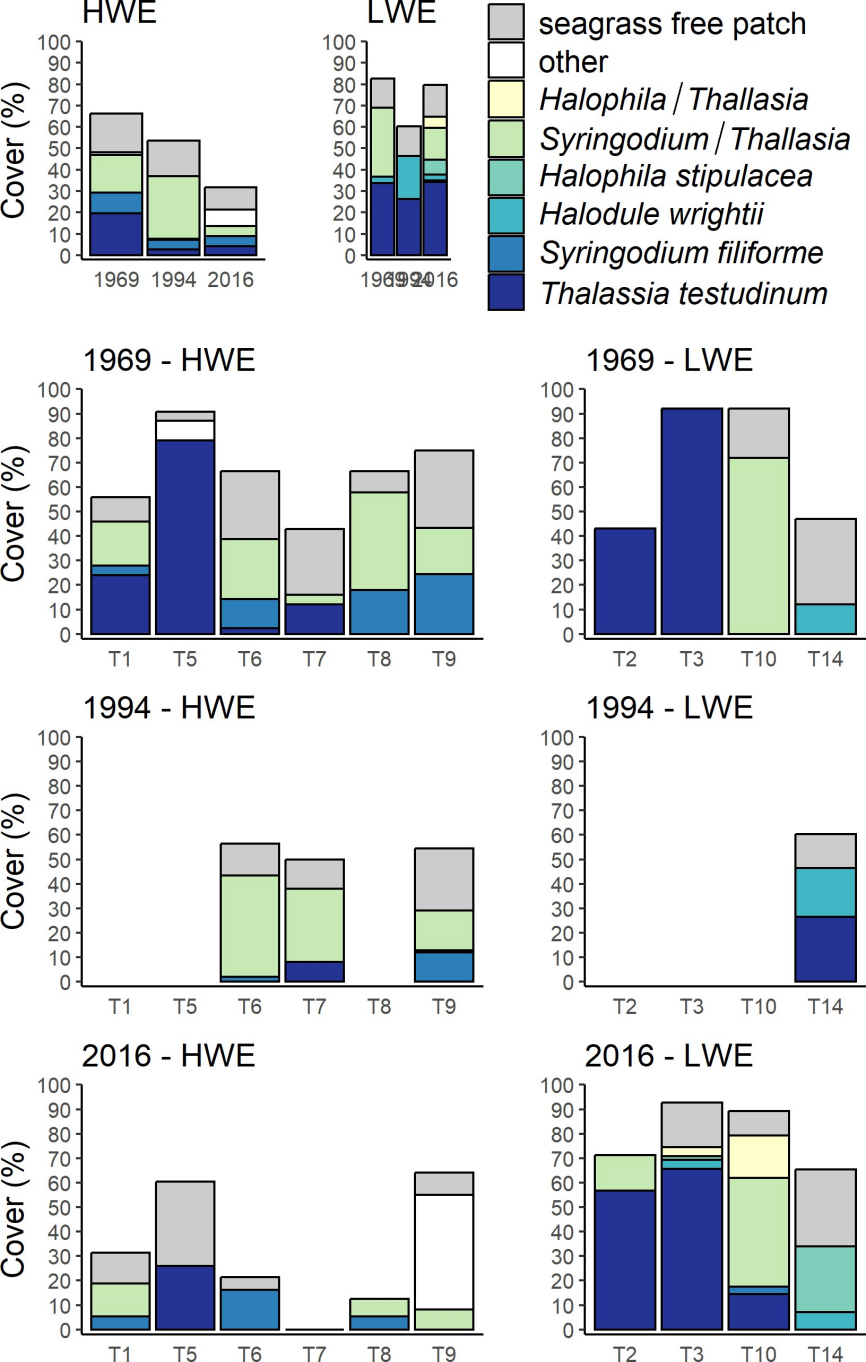
Substratum cover in seagrass beds. Mean (+ SE) % cover of transects in 6 HWE sites 4 LWE sites in 1969 and 2016, and 3 HWE sites and 1 LWE site in 1994 (top row). Cover on individual transects at HWE and LWE sites in each year (subsequent 3 rows). Height of bars indicates total cover of seagrass bed; remainder is cover of seagrass-free inshore zone (SFI). The large component “Other” in 2016 consists mainly of a mixture of *Thalassia testudinum*, *Syringodium filiforme*, and *Halodule wrightii* on Transect T9.

Total cover by seagrass along 50-m transects (Fig 2) was higher at LWE sites (mean: 69%) than at HWE sites (48.2%) in 1969. By 2016, seagrass cover had declined more than 2-fold at HWE sites (21.4%), but there was little change at LWE sites (64.7%). An extreme shift occurred at one HWE site (T5) where the elevated, sand and pebble-based seagrass bed (83% cover) that existed in 1969 had been completely scoured down to bedrock by 2016, with only a thin cover of shifting, coarse sand and *T*. *testudinum* persisting (21% cover) in sand-filled crevices. 2-way ANOVA showed a significant effect of wave exposure on total seagrass cover within transects (F_1,16_ = 11.5, P = 0.004) but no effect of year (F_1,16_ = 3.9, P = 0.065) or interaction between year and wave exposure (F_1,16_ = 2.7, P = 0.118). A similar result was obtained for total cover of a transect by the seagrass bed per se (which includes seagrass-free patches within beds). Given the unbalanced design, high variability between transects at HWE sites and the marginal test result for the effect of year, we conducted a paired-sample t-test to compare cover of the fringing seagrass bed between years (1969, 2016) within each wave exposure level. This showed a highly significant effect of Year (higher in 1969) at HWE sites (P = 0.003), but no effect at LWE sites (P = 0.771). There were no differences in cover of seagrass-free patches within seagrass beds (Fig 2) across years or wave exposures (2-way ANOVA, p > 0.47). This indicates the pronounced decrease in the extent of the bed and the total cover of seagrass at HWE sites over time is related to the expansion of the seagrass-free inshore zone (SFI), which was not evident at LWE sites.

Mean cover by monospecific *T*. *testudinum* (Fig 2) changed very little at LWE sites between 1969 (33.8%) and 2016 (34.2%) but declined by 4-fold at HWE sites (from 19.5% to 4.3%) although this difference was not statistically significant (paired samples t test, P = 0.132). In 2016 the invasive seagrass *H*. *stipulacea* was observed within 3 of the 4 LWE transects (Fig 2); at the remaining transect (T2) *H*. *stipulacea* occurred beyond our 50-m limit for analysis (at 93–100 m over the interval 0–180 m). *H*. *stipulacea* was absent from the 6 HWE transects. Seagrass cover in pure stands and mixtures was highly variable between transects (Fig 2).

### Seagrass abundance and morphology at stations

Quadrat observations at 17 stations in 1969, 1994 and 2016 provide indicators of changing conditions within contiguous seagrass beds (Fig 1). Ten of the stations (S2, S7, S8, S9, S10, S13, S16, S19, S23) were located within fringing seagrass beds and 6 stations (S3, S5, S6, S14, S24) were in ‘patches’ or ‘streaks’ or ‘cobble banks’ seaward of fringing beds. The remaining station (T12) was in an area that was part of a fringing seagrass bed in 1969 and 1994 but consisted of patches of seagrass in 2016.

**Fig 3:**
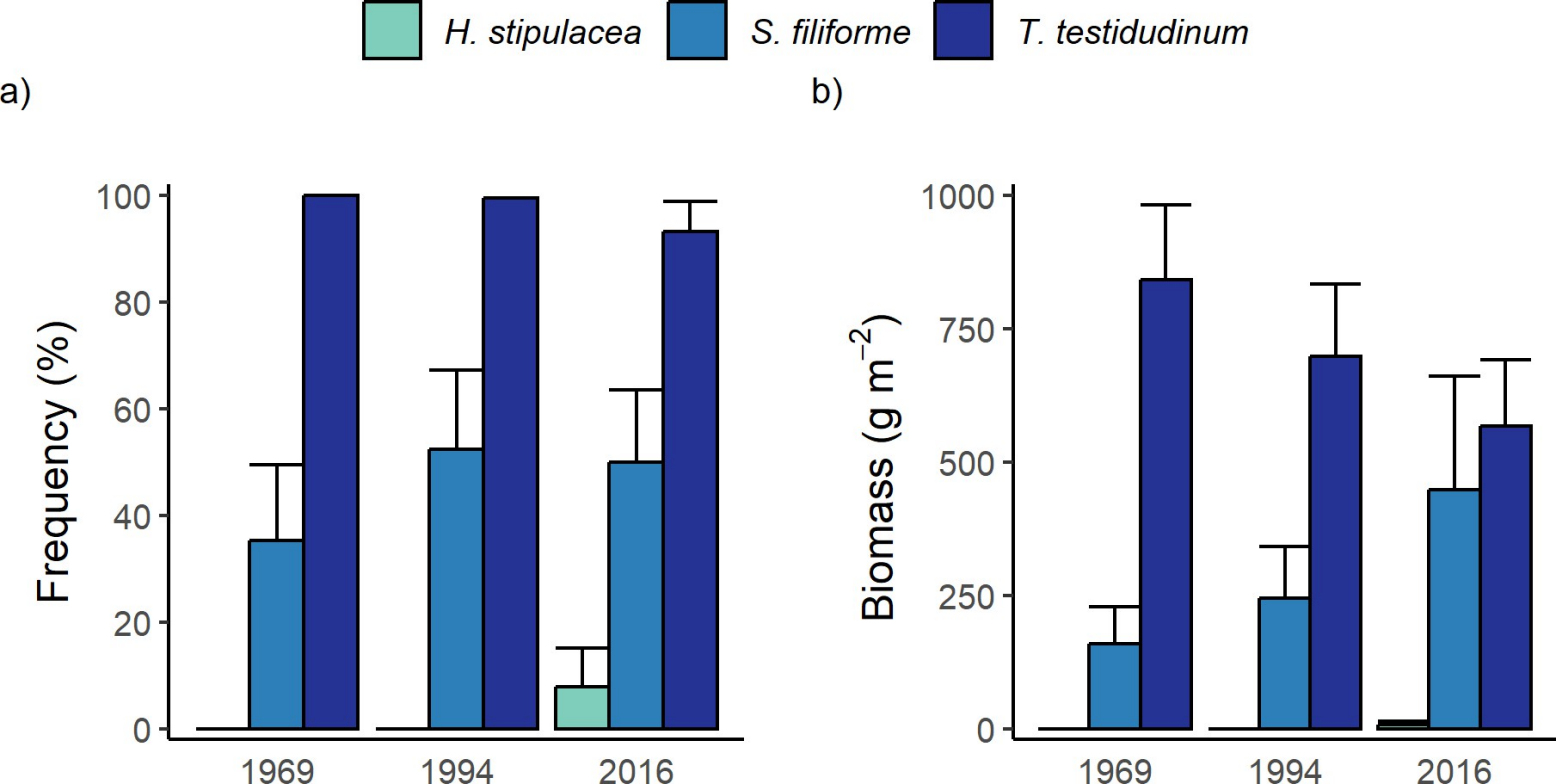
Abundance of seagrass species/groups at stations (n = 17) in 1969, 1994 and 2016. Mean (+ SE) of a) frequency of occurrence (%) and b) biomass (kg m^-2^) of *Thalassia testudinum*, *Syringodium filiforme* and *Halophila stipulacea*. (*Halodule wrightii* is not included as it occurred at very low frequencies and biomass at stations).

*Thalassia testudinum* was present at all 17 stations in all years and mean frequency (n = 12 quadrats per station) declined marginally (from 100 to 93%) between 1969 and 2016 (Fig 3A). *Syringodium filiforme* was present at 6 stations in 1969 and at 10 stations in both 1994 and 2016. Mean frequency of *S*. *filiforme* increased from 1969 (35%) to 1994 (52%) and 2016 (50%), although these changes were not statistically significant (F_2,48_, P = 0.548). *Halophila stipulacea*, accounted for the lowest recorded mean frequency (7.8%), occurring at only 2 of 17 stations in 2016. *Halodule wrightii* was not recorded in any frequency observations.

Mean leaf biomass of *T*. *testudinum* per area of bottom declined by a third (from 841 to 567 wet g m^-2^) between 1969 and 2016 (Fig 3B). In contrast, mean leaf biomass of *S*. *filiforme* increased nearly 3-fold during this time (from 159 to 448 wet g m^-2^). *H*. *stipulacea* first appeared in 2016 at 2 of the 17 stations, where it represented 59% and 5% of the local biomass but <1% (4 wet g m^-2^) of total mean biomass for all stations. *H*. *wrightii* was absent throughout, except at one station in 2016 where biomass was minimal (5.3 g m^-2^; not shown in Fig 3B). Mean total seagrass biomass changed little across the 3 sampling years (1023, 943, 1001 wet g m^-2^ respectively). These temporal changes suggest a shift in relative biomass of the two most abundant species, with similar declines and increases for *T*. *testudinum* and *S*. *filiforme* respectively. However, these changes in biomass are not statistically significant for either species (1-way ANOVA, square root transformation: *T*. *testudinum*, F_2,48_ = 1.63, P = 0.206; *S*. *filiforme*, F_2,48_ = 0.863, P = 0.448).

**Fig 4:**
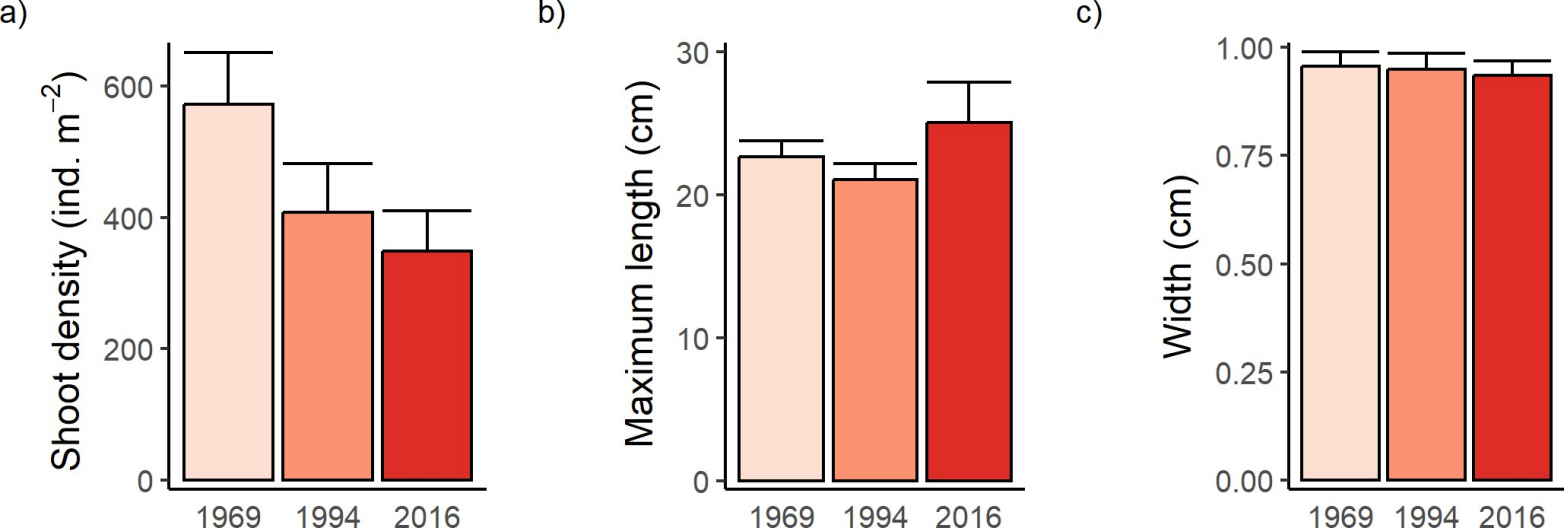
Shoot density and leaf dimensions of *Thalassia testudinum* at stations (n = 17) in 1969, 1994 and 2016. Mean (+SE) of a) shoot density (shoots m^-2^), b) maximum leaf length (cm), and c) leaf width (cm).

Mean shoot density of *T*. *testudinum* declined from 572 to 348 shoots m^-2^ between 1969 and 2016 (Fig 4A), a significant decrease to 61% of the 1969 value (1-way ANOVA, F_2,48_ = 3.7, P = 0.033; Tukey’s HSD test: P = 0.031). Mean maximum leaf length (range: 21–25 cm, (Fig 4B) did not vary significantly among years (F_2,48_ = 1.7, P = 0.201) and mean width (9.4–9.5 mm, Fig 4C) was effectively constant (F_2,48_ = 0.132, P = 0.877). Mean number of leaves per shoot also varied little between 1994 (2.8) and 2016 (2.7); leaf number was not measured in 1969. Therefore, the possible decline in biomass of *T*. *testudinum* during our study likely was related to a decrease in shoot density, rather than changes in leaf number or dimensions.

### Macroalgae and macrofauna at stations

**Fig 5:**
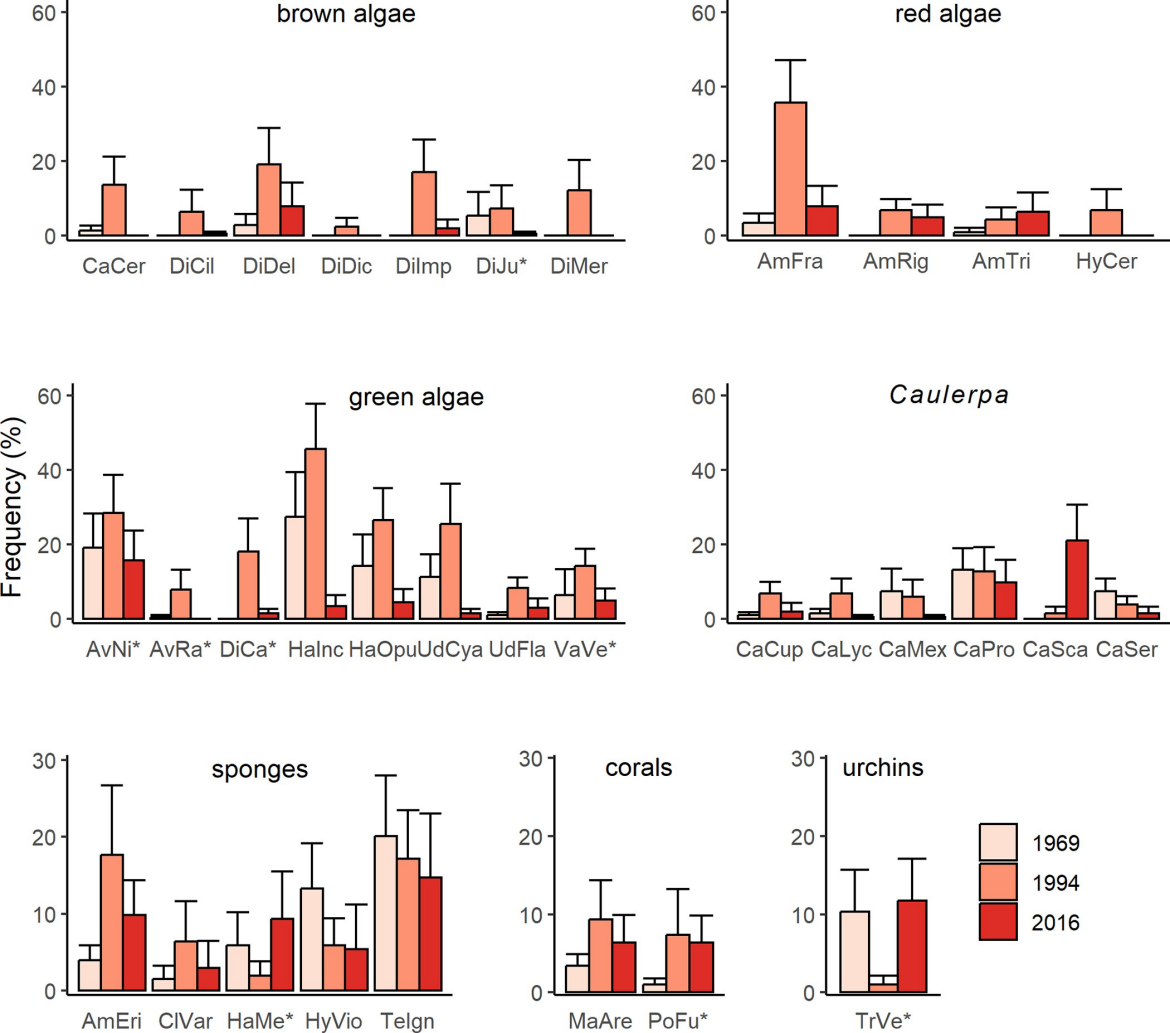
Frequency of occurrence of more common species of macroalgae and sessile macroinvertebrates, and of sea urchins, at stations (n = 17) in 1969, 1994, 2016. Mean (+ SE) frequency (%) in 12 quadrats per station. Green algae: Avrainvillea nigricans (AvNi*), Avrainvillea rawsonii (AvRa*), Dictyosphaeria cavernosa (DiCa*), *Halimeda incrassata (HaInc)*, *Halimeda opuntia (HaOpu)*, *Udotea cyathiformis (UdCya)*, *Udotea flabellum (UdFla)*, Valonia ventricosa (VaVe*); brown algae: *Canistrocarpus cervicornis (CaCer)*, *Dictyopteris delicatula (DiDel)*, Dictyopteris justii (DiJu*), *Dictyota ciliolata (DiCil)*, *Dictyota dichotoma (DiDic)*, *Dictyota implexa (DiImp)*, *Dictyota mertensii (DiMer);* Caulerpa: *Caulerpa cupressoides (CaCup)*, *Caulerpa cupressoides var*. *lycopodium (CaLyc)*, *Caulerpa mexicana (CaMex)*, *Caulerpa prolifera (CaPro)*, *Caulerpa scalpelliformis (CaSca)*, *Caulerpa sertularioides (CaSer);* red algae: *Amphiroa fragilissima (AmFra)*, *Amphiroa rigida (AmRig)*, *Amphiroa tribulus (AmTri)*, *Hypnea cervicornis (HyCer)*, coral: *Manicina areolata (MaAre)*, Porites Furcata (PoFu*); sponges: *Amphimedon erina (AmEri)*, *Cliona varians (ClVar)*, Halichondria melanodocia (HaMe*), *Hyrtios violaceus (HyVio)*, *Tedania ignis (TeIgn);* sea urchins: Tripneustes ventricosus (TrVe*).

The frequency of abundance of the more common sessile species and species groups, defined as those occurring at a mean frequency of ≥ 0.75 per 12 quadrats (6.25%) in at least one of the 17 stations in at least one year, are shown in Fig 5 (a complete list of species including the less common forms is given in S2 Table). Among macroalgae, 19 of the 25 species/groups were more frequent in 1994 (grand mean frequency, 16.5%) than in 1969 (5.0%) or 2016 (2.9%). Only the red alga *Amphiroa tribulus*, the green alga *Halimeda simulans*, and 4 of 6 *Caulerpa* spp. did not conform to this pattern. The most common *Caulerpa* species was *C*. *prolifera*, which occurred at 8, 8 and 5 stations in 1969, 1994 and 2016 respectively. *Caulerpa scapelliformis*, an invasive Indo-Pacific species, was absent at our stations in 1969, occurred at 1 station in 1994, and at 6 stations (5 with *C*. *prolifera*) in 2016.

The 2 more common corals, *Manacina areolata* and the *Porites furcata* group (*P*. *furcata* / *P*. *divaricata*), also were most frequent in 1994. There was no consistent trend with time for sponges, which as a group were common in all years. Of the 3 mobile macrofaunal species or species groups observed, only sea urchins (*Tripneustes ventricosus* group) were more common. Unlike the corals and most macroalgae, sea urchins were less frequent in 1994 (mean frequency, 0.01%) compared to 1969 (10.3%) or 2016 (11.8%). The other 2 mobile species were recorded only at low frequency in quadrats and in only 1 of the 3 sampling years (*Oreaster reticulatus*: 1.4% in 2016, *Alger gigas*: 0.98% in 1969).

### Seagrass community comparisons

**Fig 6:**
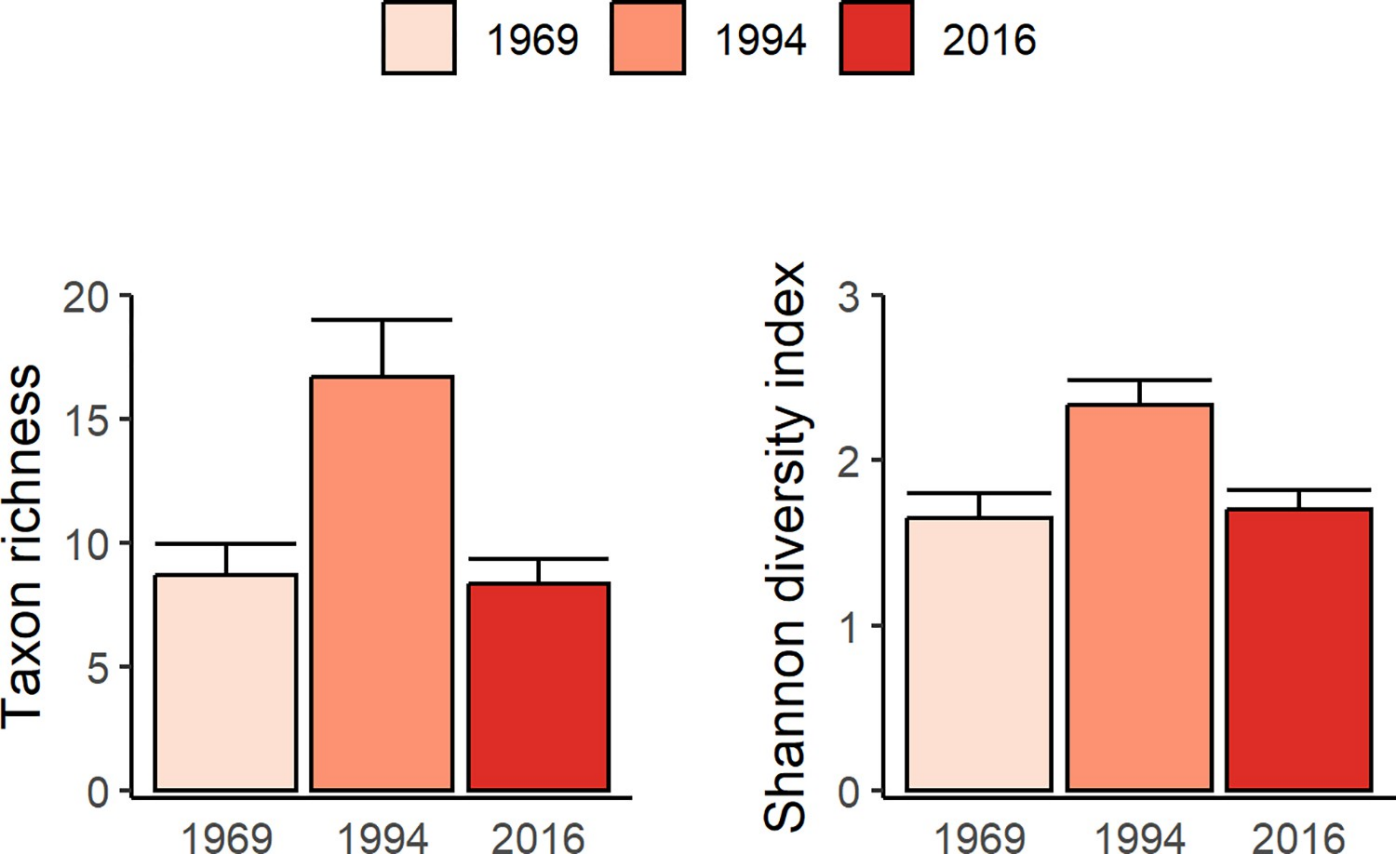
Taxon richness and diversity of the seagrass bed community. Mean + SE richness and diversity (Shannon Index) at stations (n = 17) in 1969, 1994, 2016.

**Fig 7:**
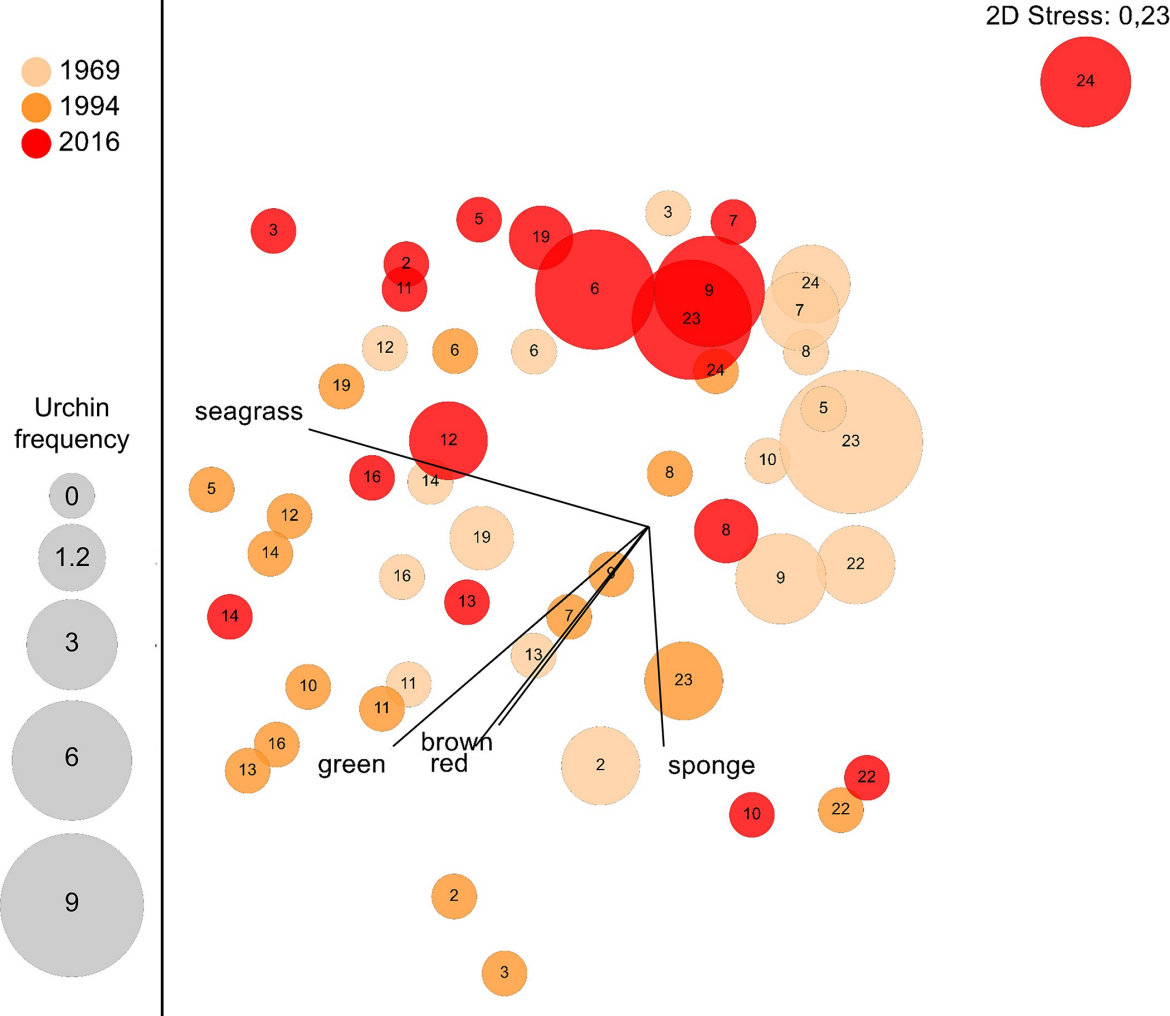
Non-metric MDS of seagrass bed community at each station and year. Points are arcsine transformed proportional frequency of occurrence measures using Bray Curtis dissimilarity matrix of square-root transformed data. Vectors (black) show correlations between ordinations for benthic communities and average frequencies of occurrence for functional groups (seagrass, brown algae, green algae and sponges) at each station and year. The size of each point indicates the frequency of sea urchins at each station and year.

Taxonomic richness and diversity (Shannon Index) of seagrass and associated macroalgae and sessile macrofauna differed significantly across the three sample years (F_2,48_ = 8.4, P <0.001 for richness; F_2,48_ = 7.3, P <0.002 for diversity) (Fig 6). In 1994, richness and diversity were significantly greater (by 2- and 1.4-fold respectively; Tukey HSD test, P <0.01) than in 1969 or 2016, when both measures did not differ (P > 0.96). The overall composition of the benthic community also differed significantly across years (PERMANOVA, P = 0.001) with a similar pattern of between-year differences: community composition was less similar between 1994 and 1969 or 2016, than between 1969 and 2016 (S3 Table). Ordination analysis (non-metric MDS) shows a broad cluster of stations in 1994 characterized by the absence (except at one site) of sea urchins (*Tripneustes ventricosus* group), in contrast to stations in both 1969 and 2016 where sea urchins occurred at 7 out of 17 stations, generally at moderate to high frequencies (Fig 7). Correlations between ordinations for benthic assemblages and frequencies of occurrence for functional groups were highest for seagrasses followed by the various macroalgal groups, and lowest for sponges.

### Extent of the seagrass-free inshore zone and erosional scarps on transects

**Fig 8:**
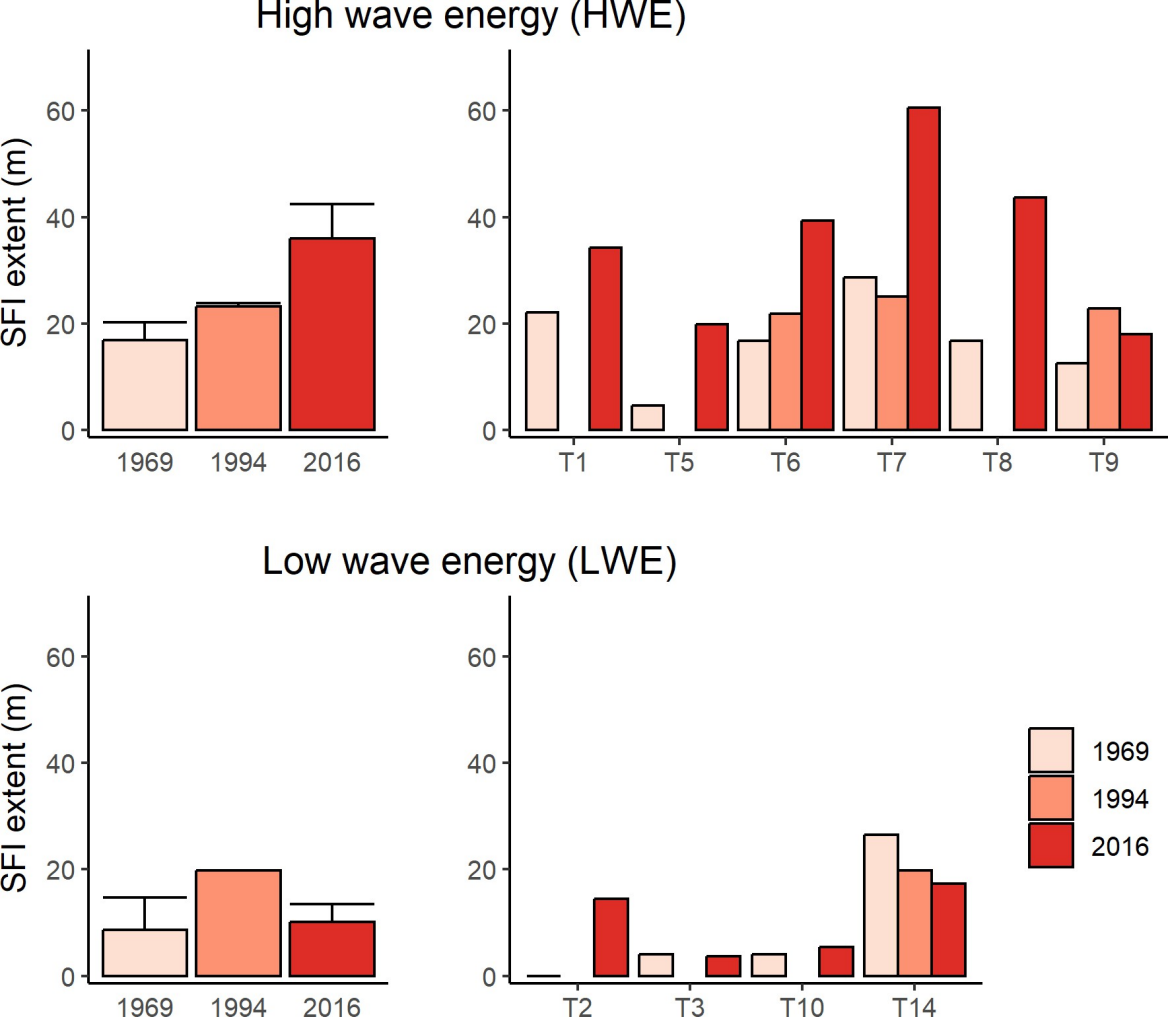
Offshore extent of the seagrass-free inshore zone (SFI, m) on transects in 1969, 1994 and 2016. Mean (± SE) extent of SFI for 6 HWE sites and 4 LWE sites in 1969 and 2016, and 3 HWE sites and 1 LWE site in 1994. Also shown (right column) is cover on replicate transects at HWE and LWE sites in each year.

The extent of the seagrass-free inshore zone (SFI) increased at all 6 HWE sites between 1969 and 2016, with a more than 2-fold increase in the average (from 16.9 to 35.9 m). In 2 of the 3 HWE transects where the SFI was measured in 1994 and SFI increased between 1969 and 2016 (T6, T7), most of this increase occurred between 1994 and 2016 (Fig 8, S2 Fig); at T9, SFI decreased between 1994 and 2016. At LWE sites, changes in SFI were generally smaller and more variable between 1969 and 2016: SFI increased measurably at one site (T2, from 0 to 14 m), decreased at another (T14, from 27 to 17 m), and was effectively stable (~ 4–5 m) at the remaining 2 sites (T3, T10), resulting in a minimal change on average (from 8.6 to 10.2 m).

2-way ANOVA showed a significant effect of year (greater in 2016) on SFI (F_1,16_ = 8.0, P = 0.012) but no effect of wave exposure (F_1,16_ = 1.2, P = 0.289) or interaction between year and wave exposure (F_1,16_ = 2.7, P = 0.119). Given the unbalanced design and high variability between transects at both LWE and HWE sites, we conducted Welch 2-sample t-tests to compare SFI between wave exposure levels in 1969 and 2016. This showed a highly significant effect of wave exposure on SFI in 2016 (t_7.2_ = -3.53, P = 0.009) but no effect in 1969 (t_4.8_ = -1.2, P = 0.287).

**Fig 9:**
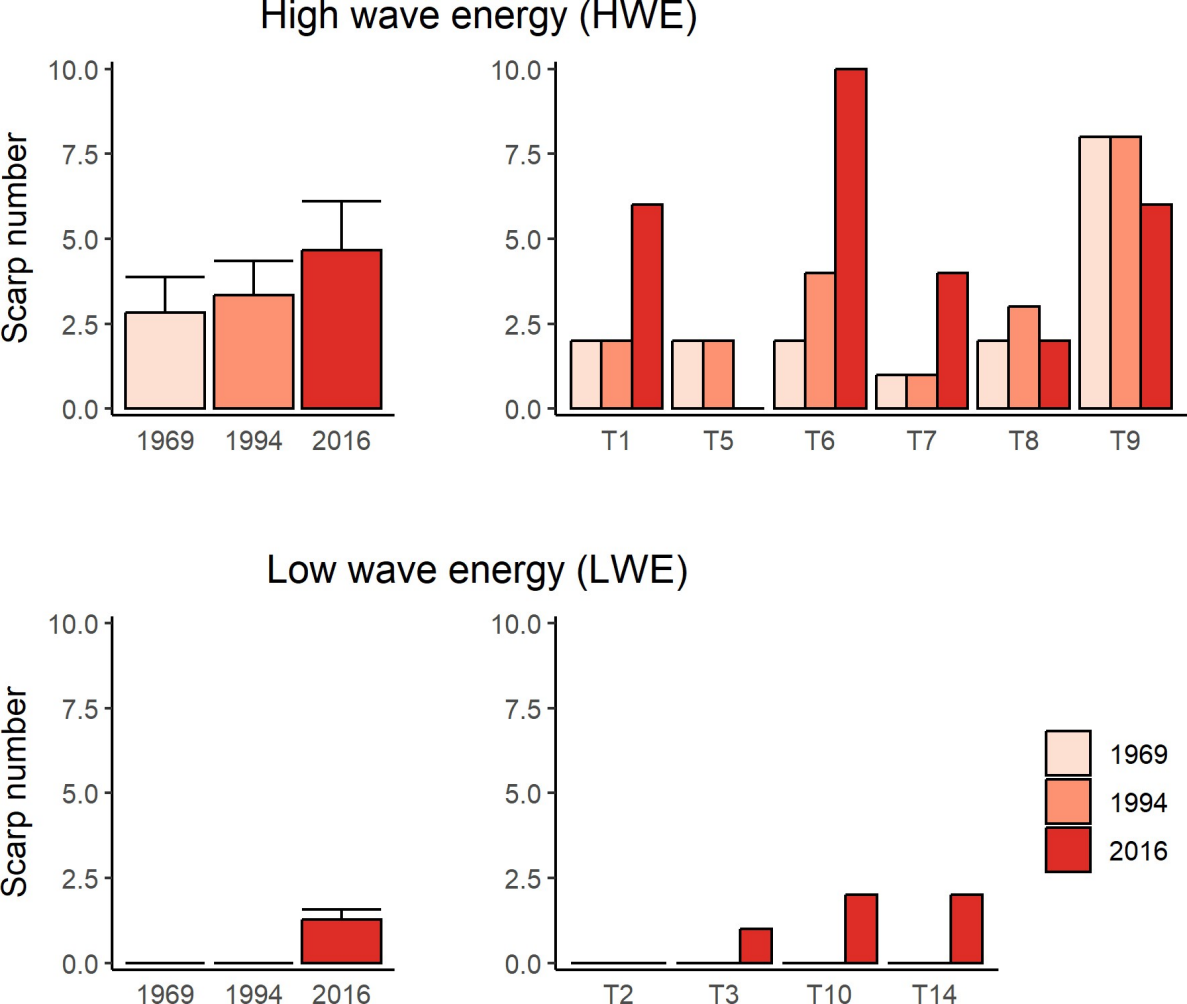
Number of scarps on transects in 1969, 1994 and 2016. Mean (± SE) scarp number for 6 HWE sites and 4 LWE sites in 1969 and 2016, and 3 HWE sites and 1 LWE site in 1994. Also shown is scarp number on replicate transects at HWE and LWE sites in each year.

No scarps were observed on transects at LWE sites in 1969 and 1994 but 1–2 scarps were present at 3 of the 4 sites in 2016. In 2016 scarps were present at on all HWE transects, except T5 where the seagrass bed had been scoured down to the underlying bed rock (Fig 9, S2 Fig). On average the number of scarps had increased 1.6-fold at HWE sites (from 2.8 to 4.6) between 1969 and 2016 (we excluded T5 from analysis as there was no possibility for scarps to form by 2016). At 3 HWE sites (T1, T6, T7), scarp number increased 3-fold between 1969 and 2016, with most or all of the increase occurring between 1994 and 2016. At the other 2 sites (T8, T9), a small decline in scarp number occurred between 1994 and 2016/2019. 2-way ANOVA showed a significant effect of wave energy (F_1,21_ = 51.1, P <0.001) and year (F_2,21_ = 5.3, P = 0.014) on scarp number, but no interaction between wave energy and year (F_2,21_ = 0.36, P = 0.702). Tukey’s HSD post hoc test showed a significant increase in scarp number between 1969 and 2016 (P = 0.019) and 1994 and 2016 (p = 0.042), but not between 1969 and 1994 (P = 0.927).

## Discussion

### Temporal and spatial context of this study

Longitudinal (multidecadal) studies of seagrass beds at individual sites in the Tropical Atlantic Bioregion have mapped changes in relative cover or extent of these beds, typically by aerial photography [22, 23, 52]. Our island-wide study, spanning 5 decades, to our knowledge, is the longest of those that record taxonomically detailed, replicated measures of abundance of seagrass and associated macroalgae and sessile invertebrates (percent cover, frequency of occurrence, biomass, density) as well as concurrent measures of seabed erosion (recession of inner bed margins, blowout frequency within beds) related to hydrodynamic forces. Although the temporal resolution is minimal, our study encompasses a period that spans a relatively undisturbed state in 1969 to an increasingly perturbed state in 2016, with a midpoint in 1994 that appears to capture a period during which biodiversity is highest but erosional features—including those indirectly related to biogenic changes such structural degradation of barrier reefs due to coral mortality—are in transition from relative stasis to an increase in magnitude. Importantly, our study spans a period of marked broad-scale environmental change in the Tropical Atlantic Bioregion, including increased sea temperature and frequency of heat waves [53], increased sea level and significant wave height [54, 55], and increased frequency and severity of strong storms [56], in addition to localized deterioration of the coastal seagrass habitat associated with eutrophication and reduced water transparency related to land based activities [21, 22, 57], dieback of *T*. *testudinum* associated with drought and hypersaline conditions [58], and direct physical disturbance by recreational or commercial activities [59].

Carriacou is a relatively small island (34 km^-2^, population ~10,000) that remains largely free of many of the localized anthropogenic factors that have caused degradation and loss of seagrass beds elsewhere in the Caribbean. It has retained a small-scale livelihood fishery (e.g., fish traps, spearfishing), in contrast to the more intensive fishing of reefs and lagoons recorded elsewhere in the Caribbean by 1985 [60]. There are no large freshwater inputs or restricted bays in Carriacou that might cause changes in seagrass beds associated with salinity fluctuations and sediment in runoff. Blooms of free-floating *Sargassum* seaweed, which had begun to impact coastal waters in Florida and much of the Caribbean after 2012 [61], had not been observed in Carriacou by end of our study in 2019. Dredging and infilling associated with construction of a large marina in Tyrell Bay in 2015–2018, likely resulted in water quality issues which, along with increased shipping and motor vessel activity, likely impacted the seagrass community of the bay and contributed to establishment of the invasive seagrass *Halophila stipulacea* [36]. However, Tyrell Bay, the most protected bay on the island, was not included in our multi-year comparisons. The changes in seagrass beds that we observed more likely are related to broader scale phenomena that affect seagrass directly or indirectly.

### Seagrass cover, composition, and morphology

There were significant losses in nearshore seagrass cover between 1969 and 2016 at HWE sites, but not LWE sites. These losses were associated with an increase of the inshore area devoid of seagrass (SFI) which doubled in average extent in transects at HWE sites. The power to detect differences in species composition of seagrass in the nearshore between years and between HWE and LWE sites was low because of high variability in composition between transects at both HWE and LWE sites and the relatively low number of sites in an unbalanced sampling design. The only clearly defined change was the appearance of the invasive seagrass *H*. *stipulacea* in 2016, exclusively at LWE sites. Based on a broader survey of *H*. *stipulacea* at Carriacou [36], which included our 2016 transect and station observations as well as those at additional sites, we found that *H*. *stipulacea* occurred primarily in large, sheltered bays along the leeward, west coast of Carriacou where it formed extensive meadows. It also occurred in smaller patches at low wave energy sites in Watering Bay on the windward, east coast and in Manchioneal Bay on the south coast.

Station records provided indicators of temporal change in species composition and abundance of seagrass, and in morphological characteristics of *T*. *testudinum*, within broadly distributed seagrass beds. *T*. *testudinum*, was the dominant species in terms of both frequency of occurrence and biomass at our stations across all three sampling years. A gradual decline in average biomass of *T*. *testudinum*, and concomitant increase in biomass of *S*. *filiforme* (although not statistically significant for either species) suggests a shift towards earlier successional stages in these beds [35, 62]. Similar successional shifts in other parts of the Caribbean have been attributed to environmental deterioration through localized anthropogenic impacts [55]. At Carriacou, however, increased intensity of wave-driven erosional processes, including increases in frequency of erosional scarps (blowouts) that re-initiate seagrass successional processes [35], may be the primary driver of such shifts.

There was no change in leaf width or length of *T*. *testudinum* among sampling years, although there was a significant decrease in shoot density. This suggests that any decline in mean biomass was associated with a decrease in shoot density rather than changes in plant morphology or in the overall occurrence of *T*. *testudinum* at the 17 stations. Leaf length and width of *T*. *testudinum* varies with environmental conditions locally and regionally, decreasing with reduced light [63, 64] or salinity [65]. In a study of *T*. *testudinum* at 3 sites across a seagrass suitability gradient in the Gulf of Mexico, mean leaf width was 4 and 5 mm in the two lower suitability sites, and 8 mm in the highest suitability site [66]. By comparison, mean leaf width at our 17 stations in ranged from 9.4 to 9.6 mm over the 3 sampling years. The constancy of total seagrass biomass regardless of composition, and large leaf width of *T*. *testudinum*, suggests that high quality habitat for seagrass at Carriacou prevailed in all three sampling years.

### Benthic macroalgae and macrofauna in seagrass habitats

In contrast to progressive changes in seagrass cover and species-specific biomass across the three sampling years, taxonomic richness and species diversity of the benthic community (seagrass and associated macroalgae and sessile macrofauna) were greatest in 1994 compared to 1969 or 2016. Also, 94% of more common species/groupings of associated of macroalgae (excluding *Caulerpa* species) and both coral taxa were most frequent in 1994 compared to 1969 and 2016, and multivariate analysis showed that community composition was less similar between 1994 and 1969 or 2016 than between 1969 and 2016. Ordination analysis showed that these patterns in seagrass community composition across years may be related to an inverse pattern in the frequency of occurrence of sea urchins (*Tripneustes ventricosus* group), which was minimal in 1994 compared to 1969 and 2016. The importance of sea urchins as major grazers of seagrass and macroalgae is well established [67, 68] and some species are known to graze coral recruits [69–71]. Sea urchins were intensively harvested in Grenada (less so in Carriacou) for export in the early 1990s, leading to the collapse and closure of the fishery by 1995 [72]. Disease or mass mortality from other causes, reported at other Tropical Atlantic locations in the 1990s [73, 74], may have contributed to the low abundance of sea urchins that we observed in 1994. Herbivorous fish also are important grazers of macroalgae in the seagrass beds [75] and increased fishing pressure [37] may have amplified the effect of decreased sea urchin abundance in seagrass beds.

The frequency of occurrence of *Caulerpa* species did not follow the trend for other macroalgal species: the highest frequency of occurrence of the more common 4 of 6 species of *Caulerpa* occurred in 1969 and 2016, when sea urchins were relatively common. Secondary metabolites in *Caulerpa* species (notably caulerpenyne) that could limit urchin grazing [76] may account in part for this discrepancy from the general inverse pattern of macroalgal and sea urchin abundance across sampling years in our study. *Caulerpa prolifera* was the most common species, except in 2016 when *C*. *scalpelliformis* [77] was more frequent. *C*. *scalpelliformis*, an Indo-Pacific species first reported in the Caribbean at Barbados in 1966 [78] and Antigua in 1967 [79], was absent at Carriacou in 1969 and rare in 1994 but had expanded to over a third of the stations by 2016, including 5 stations where it co-occurred with *C*. *prolifera*.

### Erosional features and expansion of the inshore seagrass-free zone

The greater extent of the seagrass-free inshore zone (SFI) and the higher frequency of erosional scarps and blowouts on transects across fringing seagrass beds at HWE sites compared to LWE sites are consistent with studies showing that, in the absence of significant anthropogenic disturbance, variation in structural features and cover by seagrass is related to differences in wave energy or current speed required to initiate sediment movement, combined with factors that affect erodibility of sediments [80–82].

At HWE sites, the average extent of the SFI more than doubled between 1969 and 2016 (an increase of 19 m) and for the 2 of 3 transects also measured in 1994 (T6, T7) most of the increase occurred between 1994 and 2016. As our transects began at the MLW mark, and not at a geographically fixed position, landward recession of the MLW contour due to erosion could account, at least in part, for an increase in SFI. All HWE sites are on shorelines without any man-made structures (e.g., walls or dwellings) within 50 m of shore and where there has been no attempt to reduce shoreline erosion. Cambers [83] found that beach erosion on 5 volcanic islands in the Eastern Caribbean averaged 0.65 m y^-1^ between 1985 and 2000. In Carriacou, Fitzpatrick et al. [84] estimated an erosion rate of 1 m y^-1^ between 1999 and 2006 at a sandy beach in Grand Bay (between HWE transects T8 and T9) where sand mining contributed to erosion. Assuming a conservative erosion rate of 0.5 m y^-1^ between 1969 and 2016, landward recession of the MLW contour at our HWE sites would be 23.5 m, suggesting it could account for most or all of the increase in SFI, as opposed to net loss of seagrass at the inshore margin of beds. Average landward recession of the MLW contour associated with sea level rise alone from 1969 to 2016 (9.6 cm, based on a mean of 2.04 cm y^-1^ for 1950–2010 [85]) is estimated as 1.1 m based on the slope of the shore across transects at the 6 HWE sites. In any event, it is evident that at HWE sites (with the possible exception of T9), landward migration of seagrass beds has not kept pace with an increase in accommodation space [28] over our study period.

At LWE sites, SFI increased in extent at only one site (T2) between 1969 and 2016. The shore at T2 was steeply sloping and naturally fortified with large boulders, compared to the gently sloping, mangrove-lined (T3) or sandy (T10, T14) shores at the other LWE sites, where SFI was effectively stable or decreased between 1969 and 2016. Seawalls reduce sediment supply from the shore and enhance wave reflection and surge levels [86], which likely accounts for the observed expansion of SFI at T2.

Erosional scarps occur along the inner margins of seagrass beds where they alternate with gently sloping margins of seagrass growing towards the shore (S3 Fig) [35]. Within seagrass beds “blowouts”—characterized by an erosional scarp, a seagrass-free blowout floor and a gently sloping seagrass-colonized leeward face—form during high wave energy events and their persistence is dependent on recurrent wave- or current-induced erosion at the scarp face (S3 Fig) [35, 87, 88]. Scarps and blowouts generally are absent in more protected, low wave-energy areas [35, 87, 89]. No scarps were observed at LWE sites in 1969 and 1994, but 1 or 2 scarps occurred at 3 of the 4 sites in 2016, suggesting that mean wave energy at those sites had increased sufficiently to maintain their erosion. At 3 HWE sites (T1, T6, T7), scarp number tripled between 1969 and 2016, with most of this increase occurring between 1994 and 2016. At one site (T5), the sediments were scoured down to bedrock between 1994 and 2016, removing the elevated seagrass bed and associated scarps. This is consistent with anecdotal reports by local fishers of a massive break in the barrier reef in this area in the 2000s, after which the area became more turbulent and subject to strong currents. Our findings suggest that the frequency of high wave energy events sufficient to disrupt seagrass beds, particularly those dominated by *T*. *testudinum*, increased between 1994 and 2016 compared to 1969 to 1994. This is consistent with a significant increase in Atlantic hurricane activity from 1996 to 2005 (relative to the 1950–2000 average) attributed to local sea surface warming [90].

At the remaining two HWE sites (T8, T9), both in Grand Bay, scarp number changed little over the 3 sampling years. A large increase (27 m) in SFI at T8 between 1969 and 2016 suggests that persistent wave energy at that site had increased, but that scarp-forming, high energy events affecting other HWE sites had less impact at T8, which is partially protected by a headland to the northeast. At T9, SFI and scarp number decreased between 1994 and 2019. Bottom profiles show little change in depth between 1969 and 1994, and a small increase (30–50 cm) between 1994 and 2019 (S2 Fig), suggesting that, as at T5, there had been an increase in intensity of the wave regime. The seemingly anomalous differences at T9 may be related to a plentiful supply of pebble- and cobble-sized materials from eroding banks onshore (https://www.inaturalist.org/observations/32264949), and accretion of a rubble layer, not seen at other HWE sites in 1969 [35] or in 2016.

### Impacts of reef flattening on lagoonal seagrass beds

Increases in SFI and scarp number at HWE sites and their greater magnitude compared to LWE sites, together with greater changes in these parameters between 1994 and 2016 than between 1969 and 1994, are consistent with increased wave forcing associated with degradation of offshore coral reefs between 1994 and 2016 [33, 34]. Observations on shallow reefs dominated by *Acropora palmata* at Sandy Island on the southwest coast of Carriacou show a progression from healthy *A*. *palmata* in 1969 to dead but still standing *A*. *palmata* in 1994 [91]—following the spread of white band disease in the Caribbean in the 1970s and 1980s [92]—to near complete loss of these protective structures by 2016 when we observed these reefs (S4 Fig). In a rapid assessment of the coral reefs of Carriacou in 2005, Kramer and Dowksza [37] noted extensive physical damage caused by hurricanes Ivan (2004) and Emily (2005) to many reefs along the eastern side of the island, and coral bleaching all around the island in fall 2005. These occurrences in Carriacou are consistent with the general pattern of decline in the structural complexity of Caribbean coral reefs described by Alvarez-Filip et al. [33]: a steep decline in rugosity between 1969 and 1985, followed by relative stasis until 1998, and then a resumption in decline. These authors attribute the early decline to disease-mediated mortality of the structurally dominant Acroporoids and the latest decline to bleaching events affecting the slower growing massive corals such as *Montastrea* spp that remained as the major reef builders after the Acroporoid die-off.

In Grenville Bay, Grenada, Reguero et al. [93] used wave energy models to explore erosional effects of increases in sea level and wave height between 1950 and 2008. They concluded that these factors were less important than the degradation of adjacent healthy coral reefs in explaining localized erosion within the bay. In a comparison of seagrass beds in three coral reef lagoons of the Veracruz Reef System (SW Gulf of Mexico), Terrados and Ramírez‐García [89] observed that seagrass cover was lowest, and fragmentation and blowout number highest, by a reef that had been used as a source of building materials. They attribute this to increasing wave exposure in the lagoon through a direct anthropogenic reef-flattening effect.

The stability of seagrass ecosystems, and their associated ecosystem services, including accumulation and long-term storage of carbon in associated sediments, has been highlighted as a key unknown in blue carbon science [16, 94]. Our 47-year long dataset suggests persistence of seagrass beds in Carriacou, although observations of increased blowouts and shifts in dominant seagrass species may indicate possible threat to carbon storage capacity. The availability of new accommodation space with sea level rise recently has been recognized as a potential mechanism of expansion of coastal wetlands [28] and blue carbon stores [19, 29]. Limiting development of new anthropogenic barriers to inland wetland migration or removing existing barriers could enable the persistence of coastal wetlands and associated benefits [28, 95]. Such barriers did not exist at the Carriacou HWE sites. Yet, our observations suggest that the potential of landward migration of seagrass to increase cover and enhance carbon stocks of reef-protected seagrass beds under rapidly rising sea levels in future [29] is unlikely to be realized at Carriacou, barring a significant reversal in the processes that have caused reef flattening.

## Supporting information

S1 Table Transect and station geographic information. Locations and sites (with GPS coordinates) for (A) 10 transects and (B) 17 stations sampled between 1969 and 2016. The numbering system follows that used in our paper on distribution of the invasive seagrass *Halophila stipulacea* in Carriacou (Scheibling et al. 2018), in which positions of transects and stations were numbered sequentially around the island. (This included 3 transects conducted in 2016 only and not shown here.) Also given are direction from shore, depth (m) at 50 m offshore, and relative wave exposure (low, LWE; high, HWE) for transects; and distance offshore (m), depth (m, chart datum) at sampling grid, habitat descriptions for stations, and the iNaturalist Observation Number for photographs of species and sites. Link directly to the Observation Number or append it to: https://inaturalist.org/observations/. View iNaturalist Project at: https://inaturalist.org/projects/seagrass-bed-flora-fauna-of-carriacou-grenada-2016.
(DOCX)

S2 Table Species or species groups (denoted *, see footnotes) categorized by broad taxonomic group. Presence of species or species group in at least one station (1) or none (0), number of stations out of 17 at which the species/group was recorded, and mean frequency of occurrence (%) in 12 quadrats per station across all stations in 1969, 1994 and 2016. For additional notes on occurrence and natural history of these species and associated photographic records, see iNaturalist Project https://www.inaturalist.org/projects/seagrass-bed-flora-fauna-of-carriacou-grenada-2016.
(PDF)

S3 Table Multivariate analysis of seagrass bed community composition. Results of a one factor permutational multivariate analysis of variance (PERMANOVA) based on a Bray–Curtis similarity matrix generated from arcsine sqrt transformed frequency of abundance of species/groups in stations (n = 17) in 1969, 1994 and 2016. Year is a fixed factor and all tests used 9999 permutations and unrestricted permutation of raw data and Type III partial sums of squares. Pairwise tests and average similarity are shown for communities between years.
(PDF)

S1 Fig Wave exposure for transects. Relative Exposure Index (REI) for transects at Low Wave Energy (LWE, n = 4) and High Wave Energy (HWE, n = 6) sites.
(TIFF)

S2 Fig Depth profiles for transects at 6 HWE sites and 4 LWE sites in 1969 and 2016, and 3 HWE sites and 1 LWE site in 1994. Extent of seagrass-free inshore zone shown as dashed line at start of transects. Scarps (depth measured at base and peak) shown as circles.
(TIFF)

S3 Fig Photographs illustrating blowout and scarps in seagrass beds at Carriacou. A. Typical blowout. B. Actively eroding scarp. C. Old scarp, non-eroding; new growth of seagrass on blowout floor and over scarp face. (Photographs by David Patriquin).
(PDF)

S4 Fig Photographs of *Acropora palmata*-dominated fringing reef on seaward side of Sandy Island (12.4855, -61.4829), Carriacou, illustrating the process of reef flattening between 1969 and 2016. 1969: Intact, living *A*. *palmata* reef. 1996: *A*. *palmata* entirely dead but mostly still in-place following die-off from White Band Disease in late 1970s/early 1980s; a few small colonies of a new generation of *A*. *palmata* were present. 2016: Original *A*. *palmata* framework reduced to rubble, no substantive replacement by newer colonies. (Photographs by David Patriquin.) For details of changes from 1969 to 1996, see Patriquin DG, Hunte W. Preliminary observations of the status of shallow water reefs at Sandy Island, Carriacou, Grenada. Report to the Grenada Board of Tourism and the Kido Project Environment Station, Carriacou. 1997. Available at: https://dalspace.library.dal.ca/handle/10222/82532.
(PDF)
