# Supplementary material for: Shifts in biodiversity and physical structure of seagrass beds across 5 decades at Carriacou, Grenadines
Source: PLoS One. 2024 Aug 1;19(8):e0306897. doi: 10.1371/journal.pone.0306897 (PMC11293663; doi:10.1371/journal.pone.0306897)
Supplement: S1 Table — Locations and sites (with GPS coordinates) for (A) 10 transects and (B) 17 stations sampled between 1969 and 2016. The numbering system follows that used in our paper on distribution of the invasive seagrass Halophila stipulacea in Carriacou (Scheibling et al. 2018), in which positions of transects and stations were numbered sequentially around the island. (This included 3 transects conducted in 2016 only and not shown here.) Also given are direction from shore, depth (m) at 50 m offshore, and relative wave exposure (low, LWE; high, HWE) for transects; and distance offshore (m), depth (m, chart datum) at sampling grid, habitat descriptions for stations, and the iNaturalist Observation Number for photographs of species and sites. Link directly to the Observation Number or append it to: https://inaturalist.org/observations/. View iNaturalist Project at: https://inaturalist.org/projects/seagrass-bed-flora-fauna-of-carriacou-grenada-2016. (DOCX) [file pone.0306897.s001.docx]

**S1 Table. Transect and station geographic information.** Locations and sites (with GPS coordinates) for (A) 10 transects and (B) 17 stations sampled between 1969 and 2016. The numbering system follows that used in our paper on distribution of the invasive seagrass *Halophila stipulacea* in Carriacou (Scheibling et al. 2018), in which positions of transects and stations were numbered sequentially around the island. (This included 3 transects conducted in 2016 only and not shown here.) Also given are direction from shore, depth (m) at 50 m offshore, and relative wave exposure (low, LWE; high, HWE) for transects; and distance offshore (m), depth (m, chart datum) at sampling grid, habitat descriptions for stations, and the iNaturalist Observation Number for photographs of species and sites. Link directly to the Observation Number or append it to: <https://inaturalist.org/observations/>. View iNaturalist Project at: <https://inaturalist.org/projects/seagrass-bed-flora-fauna-of-carriacou-grenada-2016>

A. Transects

| No. | Location | Coordinates | | Direction  offshore (degrees) | Depth 50 m offshore  (m) | Wave  Exposure | iNaturalist  Observation  Number |
| --- | --- | --- | --- | --- | --- | --- | --- |
| T1 | Petit Carenage Bay | | 12.526206  -61.436210 | 36 | 1.8 | HWE | [26316049](https://www.inaturalist.org/observations/26316049) |
| T2 | Watering Bay N | | 12.512881  -61.430128 | 89* | 1.3 | LWE | [34732033](https://www.inaturalist.org/observations/34732033) |
| T3 | Watering Bay S | | 12.505801  -61.426082 | 55 | 1.3 | LWE | [34806963](https://www.inaturalist.org/observations/34806963) |
| T5 | Watering/Jew Bay | | 12.500645  -61.419880 | 85 | 0.6 | HWE | [34682758](https://www.inaturalist.org/observations/34682758) |
| T6 | Jew Bay N | | 12.498028  -61.423446 | 124 | 1.9 | HWE | [26738646](https://www.inaturalist.org/observations/26738646) |
| T7 | Jew Bay S | | 12.492572  -61.425183 | 98 | 2.7 | HWE | [31339036](https://www.inaturalist.org/observations/31339036) |
| T8 | Grand Bay N | | 12.484747  -61.429249 | 119 | 1.9 | HWE | [31977917](https://www.inaturalist.org/observations/31977917) |
| T9 | Grand Bay S** | | 12.467101  -61.432452 | 97.5 | 1.4 | HWE | [148399052](https://www.inaturalist.org/observations/148399052) |
| T10 | Manchioneal Bay | | 12.448010  -61.484987 | 184 | 3.2 | LWE | [34295046](https://www.inaturalist.org/observations/34295046) |
| T14 | Hillsborough Bay | | 12.485285  -61.456774 | 313 | 1.2 | LWE | [34428737](https://www.inaturalist.org/observations/34428737) |

B. Stations

| No | Location | Coordinates | Distance offshore (m) | Depth  (m) | Habitat type | iNaturalist |
| --- | --- | --- | --- | --- | --- | --- |
| 2 | Petit Carenage | 12.526643  -61.435876 | 60 | 2 | Fringing bed | [26316049](https://www.inaturalist.org/observations/26316049) |
| 3 | Petit Carenage | 12.526862  -61.433699 | 200 | 3 | Offshore patch | [26316705](https://www.inaturalist.org/observations/26316705) |
| 5 | Watering Bay | 12.518372  -61.423202 | 750 | 3.1 | Lagoonal streaks; strong currents | [34779724](https://www.inaturalist.org/observations/34779724) |
| 6 | Grand Cay | 12.517743  -61.426857 | 375 | 2.3 | Patch just north of Grand Cay | [34775379](https://www.inaturalist.org/observations/34775379) |
| 7 | Watering Bay | 12.512812  -61.429159 | 100 | 2.5 | Fringing bed; strong currents | [34732033](https://www.inaturalist.org/observations/34732033) |
| 8 | Watering Bay S | 12.506200  -61.425711 | 40 | 1.2 | Fringing bed | [34806963](https://www.inaturalist.org/observations/34806963) |
| 9 | Watering Bay S | 12.506692  -61.424784 | 140 | 1.2 | Fringing bed, lee of patch reef | [34820281](https://www.inaturalist.org/observations/34820281) |
| 10 | Watering /Jew Bay | 12.500672  -61.419566 | 30 | 2.4*** | Fringing bed at headland | [34682758](https://www.inaturalist.org/observations/34682758) |
| 11 | Jew Bay N | 12.497265  -61.422321 | 140 | 3.3 | Fringing bed | [26738646](https://www.inaturalist.org/observations/26738646) |
| 12 | Jew Bay offshore | 12.496147  -61.420841 | 340 | 4.6 | Fringing bed, patchy | [34518037](https://www.inaturalist.org/observations/34518037) |
| 13 | Jew Bay S | 12.492453  -61.423829 | 150 | 2.8 | Fringing bed | [31339036](https://www.inaturalist.org/observations/31339036) |
| 14 | Grand Bay N | 12.483286  -61.424363 | 320 | 3.4 | Lagoonal patch | [31840688](https://www.inaturalist.org/observations/31840688) |
| 16 | Grand Bay S | 12.468669  -61.430513 | 200 | 2.7 | Fringing bed | [32206893](https://www.inaturalist.org/observations/32206893) |
| 19 | Manchioneal Bay | 12.447888  -61.485022 | 12 | 0.9 | Fringing bed | [34295046](https://www.inaturalist.org/observations/34295046) |
| 22 | L’Esterre Bay | 12.477468  -61.479154 | 140 | 0.5 | Cobble banks | [32756031](https://www.inaturalist.org/observations/32756031) |
| 23 | Hillsborough Bay | 12.485738  -61.457227 | 75 | 2.5 | Fringing bed | [34428737](https://www.inaturalist.org/observations/34428737) |
| 24 | Hillsborough Bay | 12.486810  -61.458521 | 260 | 4.5 | Extensive offshore patch | [34509592](https://www.inaturalist.org/observations/34509592) |

*Reported in Scheibling et al. 2018 in error as 181 degrees

** Transect 9 cited in Scheibling et al. 2018 located at 12.468696, -61.432335; by error it was located about 150 m north of the 1969 location. The transect was redone in 2019 at the correct location

*** Reported in error as 1.1 m in Scheibling et al. 2018
