# Supplementary material for: Shifts in biodiversity and physical structure of seagrass beds across 5 decades at Carriacou, Grenadines
Source: PLoS One. 2024 Aug 1;19(8):e0306897. doi: 10.1371/journal.pone.0306897 (PMC11293663; doi:10.1371/journal.pone.0306897)
Supplement: S2 Table — Presence of species or species group in at least one station (1) or none (0), number of stations out of 17 at which the species/group was recorded, and mean frequency of occurrence (%) in 12 quadrats per station across all stations in 1969, 1994 and 2016. For additional notes on occurrence and natural history of these species and associated photographic records, see iNaturalist Project https://www.inaturalist.org/projects/seagrass-bed-flora-fauna-of-carriacou-grenada-2016. (PDF) [file pone.0306897.s002.pdf]

S2 Table. Species or species groups (denoted *, see footnotes) categorized by broad taxonomic group. Presence at least one station (1) or none (0), number of stations out of 17 at which the species/group was recorded, and mean frequency of occurrence (%) in 12 quadrats per station across all stations in 1969, 1994 and 2016. For additional notes on occurrence and natural history of these species and associated photographic records, see iNaturalist Project https://www.inaturalist.org/projects/seagrass-bed-flora-fauna-of-carriacou-grenada-2016

Group	Species	Presence			Station No.			Frequency		
		1969	1994	2016	1969	1994	2016	1969	1994	2016
Seagrasses	*Halophila stipulacea*	0	0	1	0	0	2	0	0	7.8
Seagrasses	*Syringodium filiforme*	1	1	1	6	9	10	35.3	52.4	50
Seagrasses	*Thalassia testudinum*	1	1	1	17	17	17	100	99.5	93.2
Algae-Brown	*Canistrocarpus cervicornis*	1	1	0	2	5	0	1.5	13.8	0
Algae-Brown	*Dictyopteris delicatula*	1	1	1	2	6	4	2.9	19.1	7.8
Algae-Brown	*Dictyopteris justii**[1]	1	1	1	1	2	1	5.4	7.3	0.5
Algae-Brown	*Dictyota ciliolata*	0	1	1	0	2	1	0	6.3	0.5
Algae-Brown	*Dictyota dichotoma*	0	1	0	0	2	0	0	2.4	0
Algae-Brown	*Dictyota implexa*	0	1	1	0	6	1	0	17.2	2
Algae-Brown	*Dictyota mertensii*	0	1	0	0	4	0	0	12.3	0
Algae-Brown	*Dictyota pinnatifida*	0	1	0	0	1	0	0	2.9	0
Algae-Brown	*Padina sanctaecrucis**[2]	0	1	1	0	1	1	0	3.4	1
Algae-Brown	*Sargassum**	0	1	1	0	3	1	0	4.9	1.5
Algae-Brown	*Stypopodium zonale*	0	0	1	0	0	1	0	0	0.5
Algae-Caulerpa	*Caulerpa cupressoides*	1	1	1	2	6	1	1	6.8	2
Algae-Caulerpa	*Caulerpa cupressoides var. lycopodium*	1	1	1	2	6	1	1.5	6.8	0.5
Algae-Caulerpa	*Caulerpa mexicana*	1	1	1	3	5	1	7.3	5.9	0.5
Algae-Caulerpa	*Caulerpa prolifera*	1	1	1	8	8	5	13.3	12.8	9.8
Algae-Caulerpa	*Caulerpa racemosa*	1	1	0	1	4	0	0.5	3.4	0
Algae-Caulerpa	*Caulerpa racemosa var. macrophysa*	0	0	1	0	0	1	0	0	1
Algae-Caulerpa	*Caulerpa scalpelliformis*	0	1	1	0	1	6	0	1.5	21.1
Algae-Caulerpa	*Caulerpa sertularioides*	1	1	1	6	5	1	7.3	3.9	1.5
Algae-Caulerpa	*Caulerpa taxifolia*	1	1	0	1	1	0	1.5	2.4	0

Algae-Caulerpa	*Caulerpa verticillata*	0	1	0	0	1	0	0	1.5	0
Algae-Other Green	*Acetabularia calyculus*	0	1	0	0	1	0	0	1	0
Algae-Other Green	*Avrainvillea nigricans**	1	1	1	7	11	8	19.1	28.4	15.7
Algae-Other Green	*Avrainvillea rawsonii**	1	1	0	1	5	0	0.5	7.8	0
Algae-Other Green	*Codium isthmocladum*	0	1	0	0	4	0	0	2.9	0
Algae-Other Green	*Dictyosphaeria cavernosa**	0	1	1	0	7	2	0	18.2	1.5
Algae-Other Green	*Ernodesmis verticillata*	0	1	1	0	4	1	0	3.9	2.9
Algae-Other Green	*Halimeda incrassata*	1	1	1	6	13	3	27.4	45.6	3.4
Algae-Other Green	*Halimeda opuntia*	1	1	1	4	10	4	14.3	26.5	4.4
Algae-Other Green	*Halimeda simulans**	1	1	1	12	9	1	30.9	18.7	1.5
Algae-Other Green	*Penicillus capitatus**	1	1	1	5	6	2	4.9	5.9	1
Algae-Other Green	*Siphonocladus tropicus*	0	1	0	0	1	0	0	3.4	0
Algae-Other Green	*Udotea cyathiformis*	1	1	1	5	9	2	11.3	25.5	1.5
Algae-Other Green	*Udotea flabellum*	1	1	1	2	8	2	1	8.3	2.9
Algae-Other Green	*Ulva lactuca**	0	1	0	0	1	0	0	1	0
Algae-Other Green	*Valonia ventricosa**	1	1	1	2	11	4	6.3	14.3	4.9
Algae-Red	*Amphiroa fragilissima*	1	1	1	3	12	4	3.4	35.8	7.8
Algae-Red	*Amphiroa rigida*	0	1	1	0	7	3	0	6.8	4.9
Algae-Red	*Amphiroa tribulus*	1	1	1	1	3	2	1	4.4	6.3
Algae-Red	*Chondria littoralis*	0	1	1	0	1	2	0	1.5	2.9
Algae-Red	*Galaxaura rugosa*	1	1	1	1	4	1	0.5	4.9	3.4
Algae-Red	*Gelidiella acerosa*	0	1	1	0	1	1	0	1.5	0.5
Algae-Red	*Hypnea cervicornis*	0	1	0	0	1	0	0	6.8	0
Algae-Red	*Kallymenia perforata*	0	1	0	0	1	0	0	1	0
Algae-Red	*Tricleocarpa fragilis*	0	1	1	0	5	2	0	4.9	1.5
Algae-Red	*Yuzurua poiteaui*	0	1	0	0	2	0	0	4.4	0
Corals	*Amphimedon erina*	1	1	1	5	5	7	3.9	17.7	9.8
Corals	*Favia fragum*	0	1	0	0	1	0	0	0.5	0
Corals	*Manicina areolata*	1	1	1	6	8	5	3.4	9.3	6.3
Corals	*Porites astreoides*	0	0	1	0	0	1	0	0	0.5
Corals	*Porites furcata**	1	1	1	2	4	5	1	7.3	6.3
Corals	*Porites porites*	1	0	1	2	0	1	4.4	0	1
Corals	*Siderastrea radians*	1	1	0	2	6	0	1.5	5.4	0
Echinoid	*Tripneustes ventricosus**	1	1	1	7	1	7	10.3	0.01	11.8
Gorgonia	*Briaerium asbestinum*	1	1	0	1	2	0	5.4	1.5	0
Gorgonia	*Gorgonia ventalina*	0	1	0	0	1	0	0	0.5	0

Gorgonia	*Muriceopsis sulphurea*	0	1	0	0	4	0	0	5.4	0
Gorgonia	*Pterogorgia citrina*	1	1	0	1	1	0	0.5	0.5	0
Sponges	*Amphimedon compressa*	1	1	1	2	2	1	1	1.5	0.5
Sponges	*Aplysina fulva*	0	1	1	0	1	1	0	1	1.5
Sponges	*Cliona varians*	1	1	1	1	2	1	1.5	6.3	2.9
Sponges	*Halichondria melanodocia**	1	1	1	4	2	5	5.9	2	9.3
Sponges	*Hyrtios violaceus*	1	1	1	7	4	2	13.3	5.9	5.4
Sponges	*Tedania ignis*	1	1	1	8	9	5	20.1	17.2	14.7

Species groups:

1. *Dictyopteris justii*: Dictyopteris justii* &/or *D. jolyana*
2. *Padina sanctaecrucis*: Padina sanctae-crucis* &/or *P. gymnospora*
3. *Sargassum*: S. hystrix &/or S. platycarpum, possibly other species.*
4. *Avrainvillea nigricans*: A. nigricans an*d/or *A. longicaulis* and possibly any one or more of 6 other species of *Avrainvillea* in the West Indies with stipes and paddle- or fan-shaped blades and occurring in the Lesser Antilles (*A. ascarifolia, A. elliotti, A. fulva, A levis, A. mazei, A. silvana).* See: Littler DS, Littler DM. Caribbean Reef Plants. OffShore Graphics, Washington, D.C. 2000. ISBN 0-9678901-0-1.
5. *Avrainvillea rawsonii*: A. rawsonii and/or A. digitata* (finger to club shaped blades interwoven/in mats)
6. *Dictyosphaeria cavernosa*: Dictyosphaeria cavernosa** &/or *D. ocellata*
7. *Halimeda simulans*:* May have included some *H. tuna*
8. *Penicillus capitatus*: Penicillus capitatus* &/or P. *dumetosus*
9. *Ulva lactuca*: Ulva lactuca &/or U. fasciata*
10. *Valonia ventricosa*: Valonia ventricosa* &/or *V. macrophysa*
11. *Porites furcata*: Porites furcata* &/or *P. divaricata*
12. *Tripneustes ventricosus*: Tripneustes ventricosus &/or Lytechinus variegatus;* most were *T. ventricosus.*
