# Supplementary material for: Shifts in biodiversity and physical structure of seagrass beds across 5 decades at Carriacou, Grenadines
Source: PLoS One. 2024 Aug 1;19(8):e0306897. doi: 10.1371/journal.pone.0306897 (PMC11293663; doi:10.1371/journal.pone.0306897)
Supplement: S3 Table — Results of a one factor permutational multivariate analysis of variance (PERMANOVA) based on a Bray–Curtis similarity matrix generated from arcsine sqrt transformed frequency of abundance of species/groups in stations (n = 17) in 1969, 1994 and 2016. Year is a fixed factor and all tests used 9999 permutations and unrestricted permutation of raw data and Type III partial sums of squares. Pairwise tests and average similarity are shown for communities between years. (PDF) [file pone.0306897.s003.pdf]

S3 Table. Multivariate analysis of seagrass bed community composition. Results of a one factor permutational multivariate analysis of variance (PERMANOVA) based on a Bray–Curtis similarity matrix generated from arcsine sqrt transformed frequency of abundance of species/groups in stations (n = 17) in 1969, 1994 and 2016. Year is a fixed factor and all tests used 9999 permutations and unrestricted permutation of raw data and Type III partial sums of squares. Pairwise tests and average similarity are shown for communities between years.

Source	df	MS	Pseudo-F	P(perm)
Year	2	6722	3.33	0.001
Residual	48	2019		
Pairwise groups	Average similarity %		t	P(perm)
1969, 1994	34.2		1.911	0.003
1969, 2016	35.2		1.567	0.014
1994, 2016	30.9		1.963	0.001
