# Supplementary figures and images for: Shifts in biodiversity and physical structure of seagrass beds across 5 decades at Carriacou, Grenadines
Source: PLoS One. 2024 Aug 1;19(8):e0306897. doi: 10.1371/journal.pone.0306897 (PMC11293663; doi:10.1371/journal.pone.0306897)

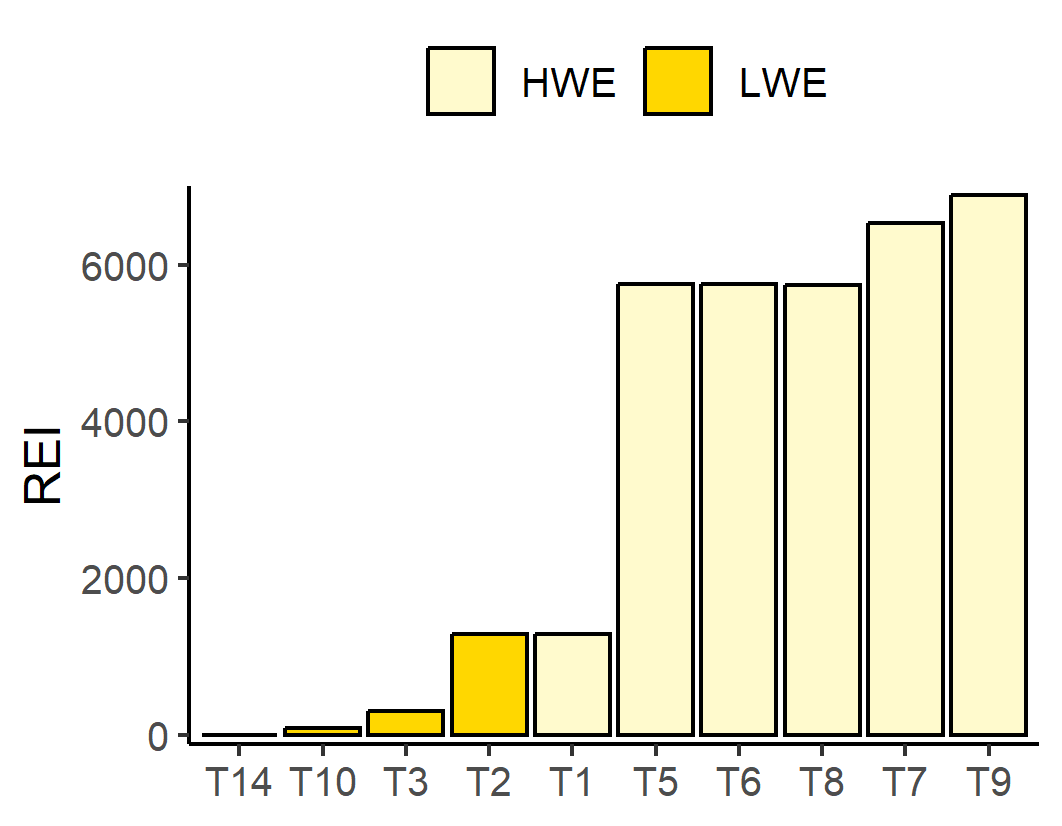

Supplement: S1 Fig — Relative Exposure Index (REI) for transects at Low Wave Energy (LWE, n = 4) and High Wave Energy (HWE, n = 6) sites. (TIFF) [file pone.0306897.s004.tiff]

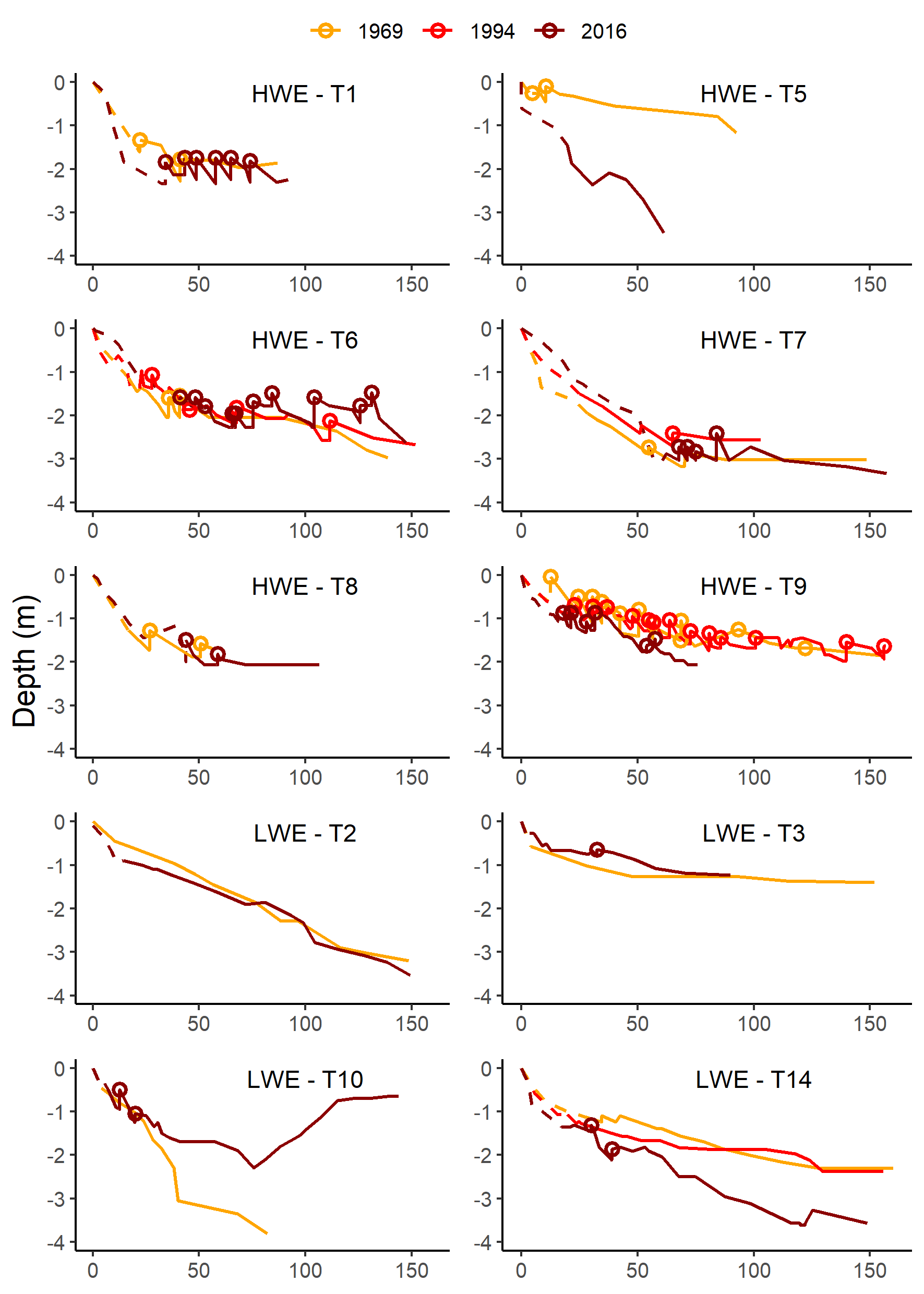

Supplement: S2 Fig — Extent of seagrass-free inshore zone shown as dashed line at start of transects. Scarps (depth measured at base and peak) shown as circles. (TIFF) [file pone.0306897.s005.tiff]

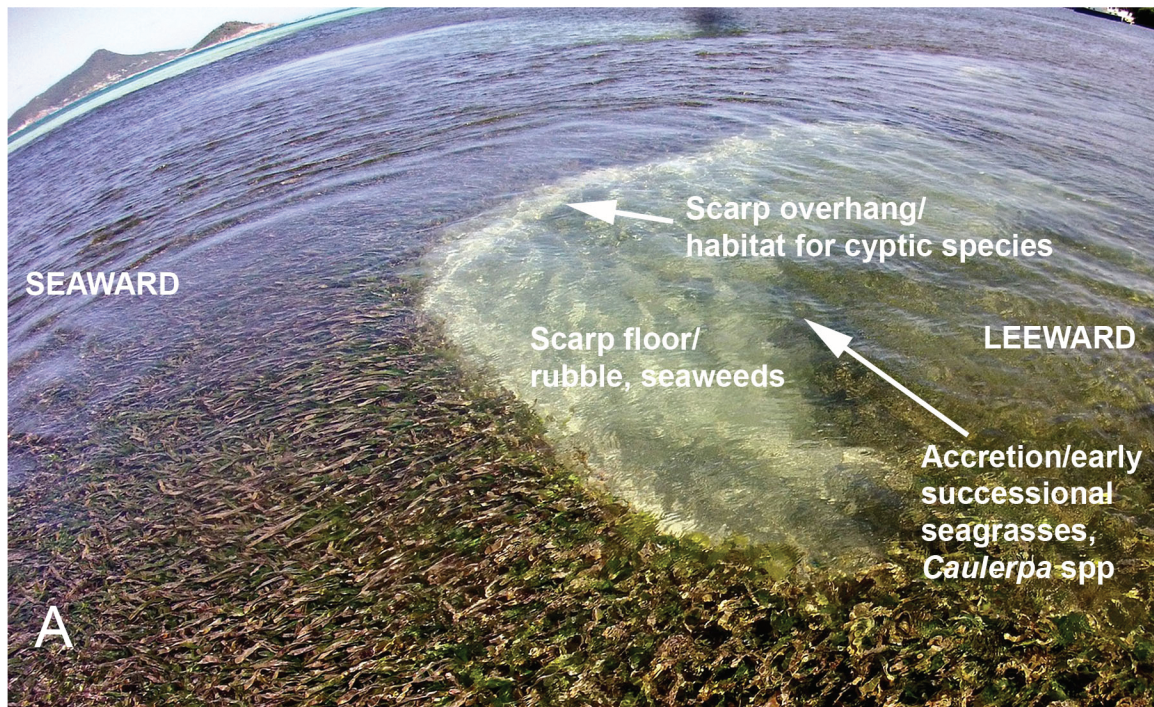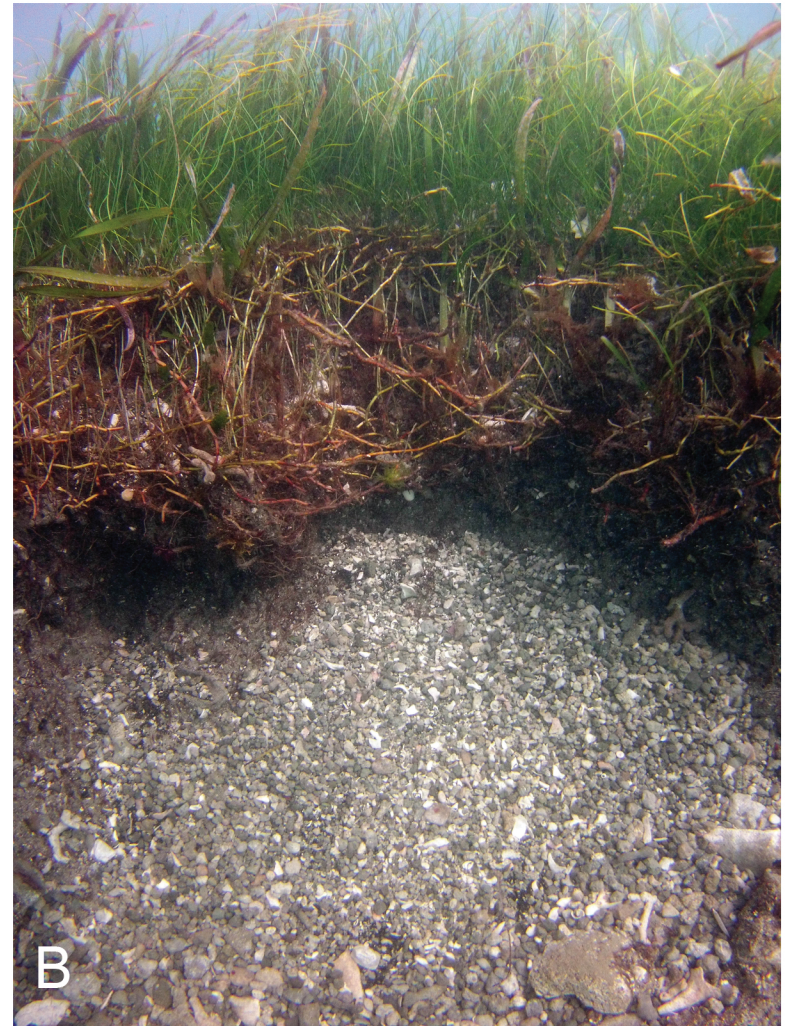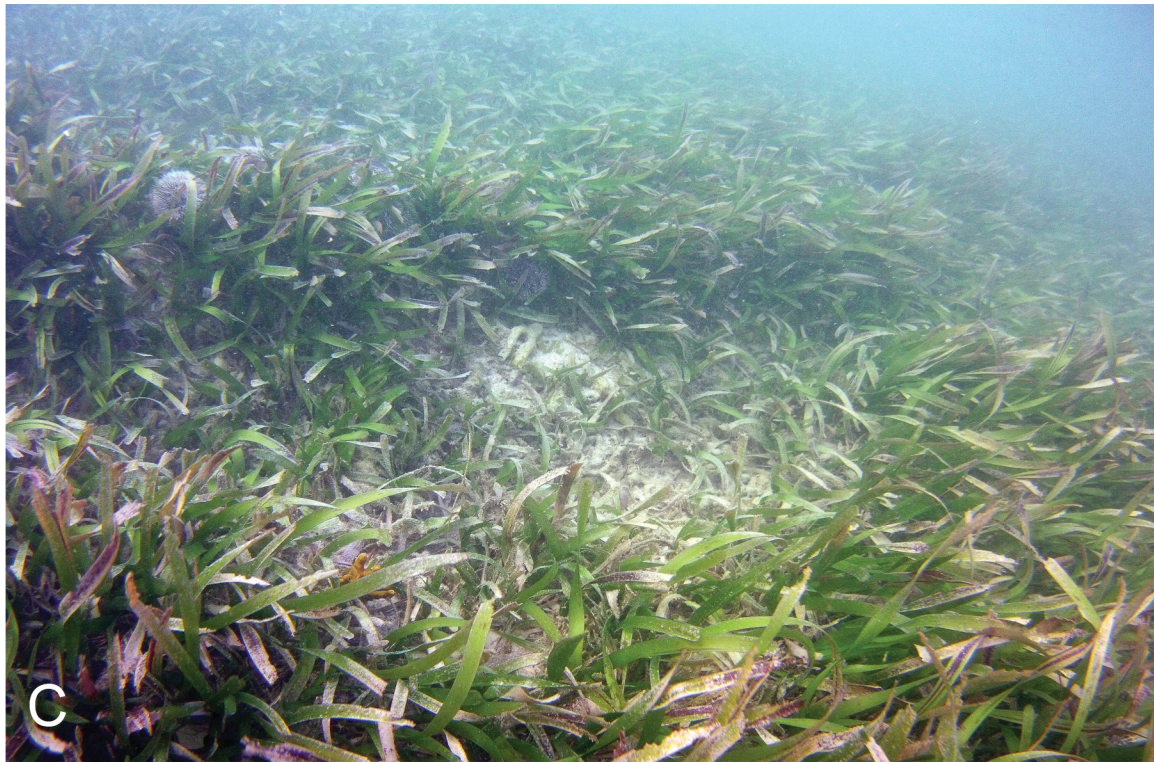

Supplement: S3 Fig — A. Typical blowout. B. Actively eroding scarp. C. Old scarp, non-eroding; new growth of seagrass on blowout floor and over scarp face. (Photographs by David Patriquin). (PDF) [file pone.0306897.s006.pdf]

1969

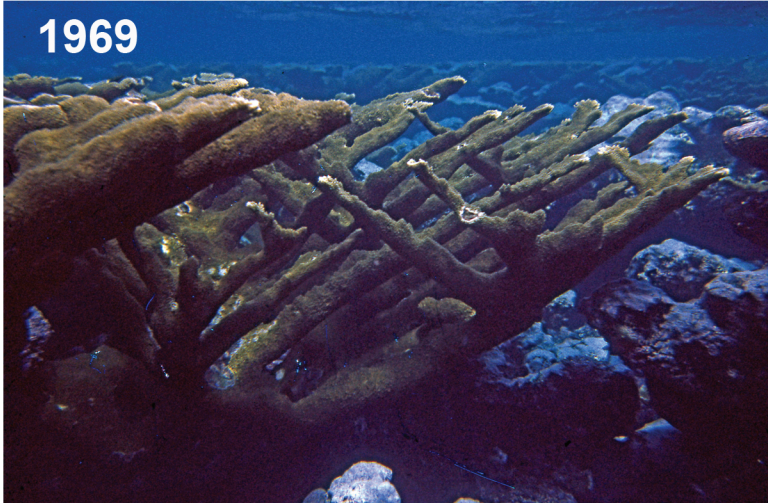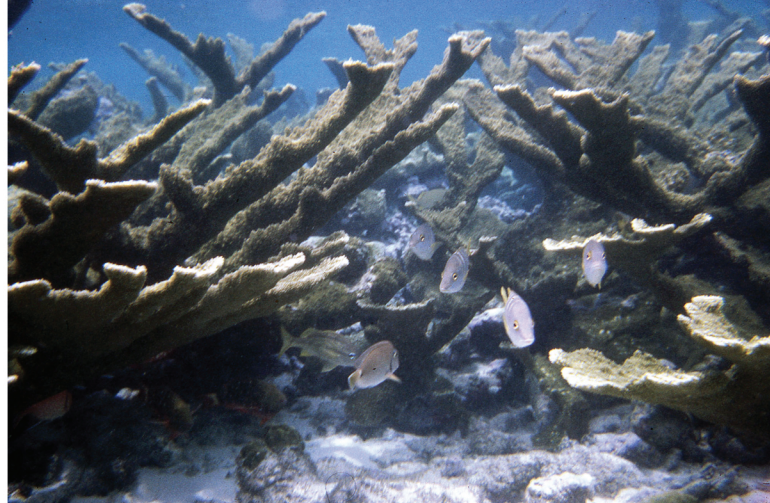

1996

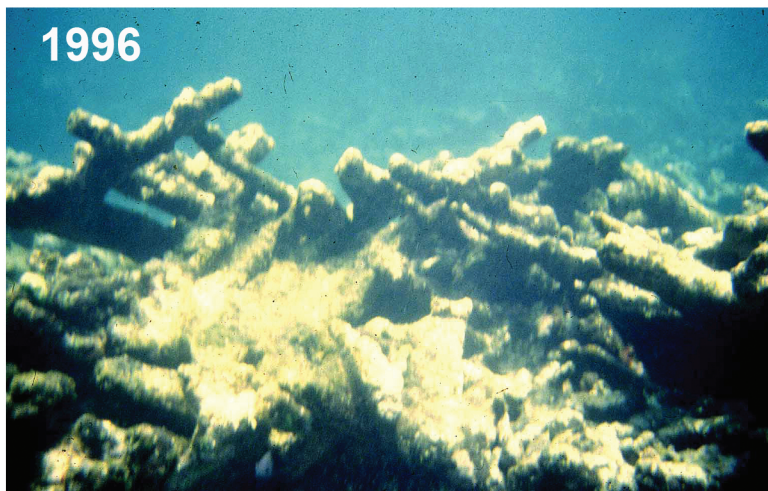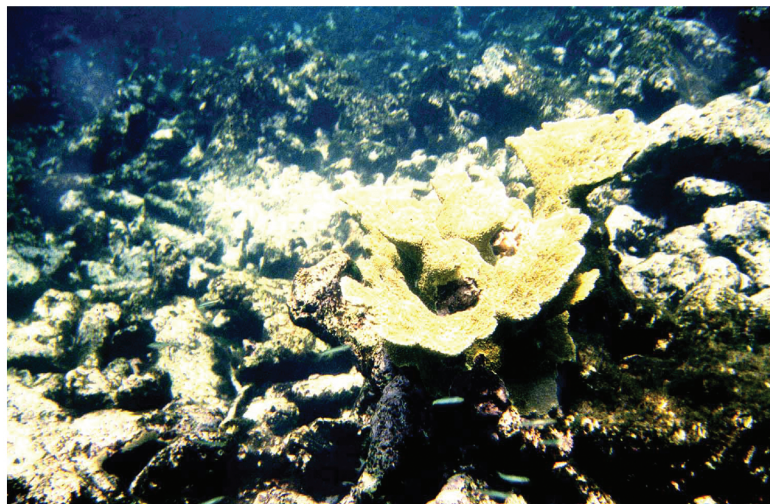

2016

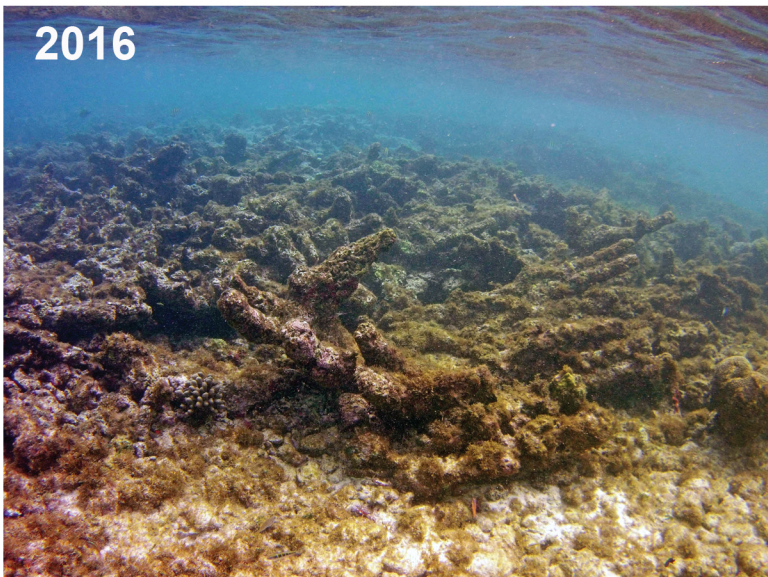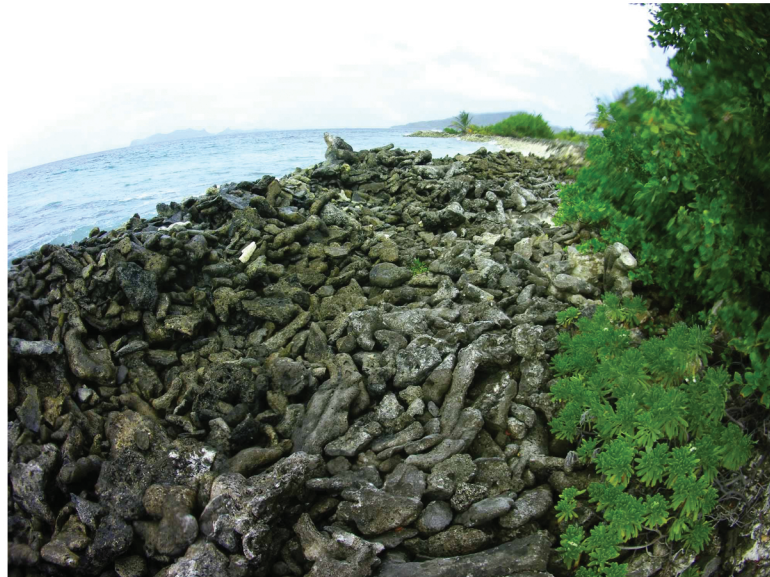

Supplement: S4 Fig — 1969: Intact, living A. palmata reef. 1996: A. palmata entirely dead but mostly still in-place following die-off from White Band Disease in late 1970s/early 1980s; a few small colonies of a new generation of A. palmata were present. 2016: Original A. palmata framework reduced to rubble, no substantive replacement by newer colonies. (Photographs by David Patriquin.) For details of changes from 1969 to 1996, see Patriquin DG, Hunte W. Preliminary observations of the status of shallow water reefs at Sandy Island, Carriacou, Grenada. Report to the Grenada Board of Tourism and the Kido Project Environment Station, Carriacou. 1997. Available at: https://dalspace.library.dal.ca/handle/10222/82532. (PDF) [file pone.0306897.s007.pdf]
